# Peroxisome proliferator-activated receptor gamma (PPARγ) as a mechano-metabolic transducer: coordinating lipid homeostasis through mechanical cues

**DOI:** 10.1186/s43556-026-00544-y

**Published:** 2026-08-12

**Authors:** Ming-Yue Zhong, Shu-Ya Yang, Wen-Hui Xu, Ying-Kang Zhang, Jun Zhao

**Affiliations:** Laboratory for Corneal Tissue Engineering, College of Marine Life Sciences, Ocean University of China, Qingdao, Shandong Province 266003 People’s Republic of China

**Keywords:** PPARγ, Metabolic diseases, Mechano-metabolic transducer, Mechanical microenvironment, Lipid metabolism

## Abstract

The pathogenesis of prevalent metabolic diseases such as obesity, atherosclerosis, metabolic dysfunction-associated steatotic liver disease, and diabetes is intricately linked to dysregulated lipid metabolism. Peroxisome proliferator-activated receptor gamma (PPARγ) is a key transcriptional regulator of lipid homeostasis and a well‑researched therapeutic target. Although biochemical signaling pathways have been the traditional focus, recent studies now highlight the mechanical microenvironment (matrix stiffness, fluid shear stress, and tensile strain) as a pivotal physical metabolism regulator. However, how mechanical signals integrate with PPARγ to control lipid metabolism across tissues and diseases remains poorly defined. This review details the molecular mechanisms by which mechanical cues influence PPARγ expression, activity, and post‑translational modifications, focusing on Yes-associated protein (YAP)/transcriptional coactivator with PDZ-binding motif (TAZ), neural precursor cell expressed developmentally down-regulated protein 4 (NEDD4)-mediated ubiquitination, and protein kinase Cα (PKCα)-extracellular signal-regulated kinase (ERK) pathways. We further explore the critical role of PPARγ in mechano‑metabolic coupling in adipose tissue, liver, and vasculature. Under normal physiological conditions, mechanical loading suppresses PPARγ to promote osteogenesis and vascular homeostasis; under pathological conditions, aberrant signals and PPARγ dysfunction establish a vicious cycle of “mechanical imbalance–metabolic disorder–tissue remodeling.” This review suggests that PPARγ may function as a mechano‑metabolic downstream transcriptional transducer linking the mechanical microenvironment to metabolic reprogramming, thereby offering a novel theoretical framework and translational perspective for the physical intervention and targeted therapy of common metabolic diseases.

## Introduction

Lipid metabolism disorders, including obesity, type 2 diabetes (T2D), metabolic dysfunction-associated steatotic liver disease (MASLD), and atherosclerotic cardiovascular disease, are major global public health challenges [1–3]. The traditional research paradigm has focused on deciphering pathogenesis through biochemical signals, such as hormones, nutrients, and inflammatory factors. However, the physical microenvironment, particularly the mechanical signals, constitutes a critical regulatory dimension that substantially influences disease progression [4]. Mechanical signals, such as fluid shear stress, extracellular matrix (ECM) stiffness, and cellular tension, are fundamental inputs through which cells perceive their physical surroundings. Mechanotransduction is classically defined as the process by which cells convert these mechanical signals into a biochemical response [5]. Mechanobiological research has revealed that these forces shape tissue morphology and directly regulate cellular differentiation, metabolism, and inflammatory states via a mechanosensing**–**signal transduction**–**phenotypic regulation axis [6–9]. However, it is unclear whether the mechanical alterations observed in metabolic diseases, including tissue stiffening (e.g., in a fibrotic liver or thickened vasculature) and disturbed blood flow (e.g., oscillatory shear stress [OSS] at arterial bifurcations), are passive consequences or active drivers of metabolic dysfunction.

The role of Peroxisome proliferator-activated receptor gamma (PPARγ), a master regulator of lipid metabolism and glucose homeostasis, is crucial [10–12]. Although PPARγ responds to chemical ligands, such as fatty acids, its expression and activity are also precisely modulated by mechanical signals across various cell types [13]. For example, matrix stiffness suppresses PPARγ via the Yes-associated protein (YAP)/transcriptional coactivator with PDZ-binding motif (TAZ) pathway, promoting osteogenic over adipogenic differentiation [14, 15]. Similarly, hemodynamic abnormalities downregulate PPARγ through protein kinase Cα (PKCα)-extracellular signal-regulated kinase (ERK) or neural precursor cell expressed developmentally downregulated protein 4 (NEDD4)-mediated ubiquitination, thereby promoting vascular inflammation and fibrosis [16, 17]. These findings suggest that PPARγ functions not merely as an isolated metabolic regulator, but also as a molecular hub that integrates upstream mechanotransduction signals. Although PPARγ does not directly sense mechanical forces (unlike Piezo1 or integrins), it acts as a downstream transcriptional transducer that converts diverse mechanical inputs into durable changes in lipid metabolism. However, the mechanical signal-PPARγ-lipid metabolism axis remains poorly integrated. Commonalities and specificities across tissues, precise upstream mechano-sensing mechanisms, and downstream metabolic consequences require further systematic elucidation. Existing reviews have extensively covered PPARγ as a metabolic regulator in response to biochemical cues, or separately described mechanotransduction pathways in cell differentiation and disease [4, 18]. However, a systematic integration of mechanical signals with PPARγ-driven lipid metabolism is currently lacking. Here, we propose a novel conceptual framework in which PPARγ functions as a “mechano-metabolic transducer,” representing a molecular node that integrates upstream mechanosignals from matrix stiffness, fluid shear stress, and tensile strain, and converts them into transcriptional programs controlling lipid homeostasis. This perspective distinguishes our review from previous work by explicitly linking the mechanical microenvironment to PPARγ activity, explaining how aberrant mechanics establish a vicious cycle of metabolic dysregulation and tissue remodeling.

Accordingly, this review systematically outlines the molecular mechanisms by which mechanical signals regulate PPARγ expression, activity, and post‑translational modifications to influence lipid metabolism. The review covers mechanosensing pathways that converge on PPARγ, the biology of PPARγ as a mechano‑metabolic transducer, detailed molecular transduction mechanisms, role of the mechano‑PPARγ axis in physiological and pathological lipid homeostasis, and therapeutic implications including drugs and physical interventions. The review concludes with future perspectives. This integrated framework provides a novel theoretical foundation for physical intervention strategies and targeted therapies.

## Mechanical cues and cellular mechanosensing

Mechanical forces are fundamental drivers of formation and function in organisms. Specialized mechanosensory proteins, including mechano-activated ion channels, enable cells to transduce extracellular mechanical stimuli into intracellular biological activities. Mechanical forces are fundamental regulators of cell fate, tissue development, and homeostasis [19, 20]. This process, termed mechanotransduction, underpins a diverse range of cellular events, from proliferation and differentiation to tissue development and homeostasis. In addition to shaping cellular and tissue morphology, mechanical forces regulate gene transcription via cytoskeletal remodeling and alterations in adhesion structures. This cascade influences cell fate and provides a framework for understanding tissue adaptation and disease pathogenesis driven by aberrant mechanical signals [21].

### Types of mechanical signals

Mechanical signals serve as the primary inputs through which cells perceive the physical properties of their microenvironments. These signals are diverse and intricately linked to physiological functions [19]. Hemodynamic shear stress is a key mechanical stimulus for vascular endothelial cells (ECs). This fluid mechanical force, generated by blood flow, is sensed by various mechanosensors on ECs. For example, OSS, which is prevalent in atherosclerosis-prone regions, induces an inflammatory response in ECs, whereas high laminar shear stress promotes vascular smooth muscle cell (VSMCs) differentiation into an anti-atherosclerotic phenotype and reduces the atherosclerotic lesion size [22–26]. In vitro and in vivo studies further show that YAP/TAZ are activated and translocated to the nucleus in vascular ECs exposed to OSS, but not under laminar shear stress, thereby promoting atherosclerosis progression [25].

The stiffness and topography of the ECM constitute crucial mechanical signals. During MASLD progression, ECM remodeling and collagen deposition increase tissue stiffness [27], which can induce functional impairment and DNA damage in hepatocytes via the Piezo1-ERK1/2 pathway. Increased matrix stiffness promotes fibrosis by modulating fibroblast activation and differentiation [28, 29]. Integrin-mediated mechanotransduction in response to matrix stiffness influences cell proliferation, survival, and migration, particularly in pathological contexts, such as fibrosis and cancer [30]. These diverse mechanical signals are transduced into biochemical cues via distinct sensing mechanisms that contribute to tissue homeostasis and disease pathogenesis.

The diversity of mechanical signals lies in their spatio-temporal characteristics. Dynamic signals, such as cyclic stretching, exert markedly different regulatory effects on cells than static signals, such as sustained pressure. For example, vascular ECs align perpendicularly to the direction of cyclic stretching, and sustained pressure primarily modulates proliferation and apoptosis [31, 32]. Additionally, the magnitude and frequency of mechanical signals determine cellular responses. Low-magnitude vibration (0.3 g, 90 Hz), but not high-magnitude vibration, suppresses adipogenic gene expression in ovariectomized mice, thereby protecting musculoskeletal integrity and mitigating adipose phenotypes [33, 34]. Collectively, these findings indicate that the type, properties, and tissue-specific context of mechanical signals jointly shape the cellular mechanoresponse network, offering a multi-dimensional perspective for understanding mechanical regulation in physiology and disease.

### Cellular mechanisms of mechanosensing

The cellular perception of mechanical signals relies on the coordinated action of plasma membrane receptors, the cytoskeleton, and cell-ECM or cell–cell junctions. Mechanosensitive ion channels are key plasma membrane components that sense mechanical forces. Among these, the non-selective cation channel, Piezo1,a prototypical mechanosensitive cation channel opens in response to mechanical stretching to mediate Ca^2^⁺ influx, thereby regulating processes, such as cell differentiation and metabolism [35, 36]. The cytoskeleton serves as the structural basis for mechanical signal transduction. The dynamic rearrangement of actin filaments, microtubules, and intermediate filaments converts mechanical forces into biochemical signals. For instance, tension changes in actin stress fibers can activate the focal adhesion kinase (FAK)-proto-oncogene tyrosine-protein kinase Src (Src) signaling pathway, promoting cell proliferation and migration [37].

Cell-ECM junctions transmit mechanical signals intracellularly via integrin receptors. Upon binding to ECM ligands, such as fibronectin or collagen, integrins trigger downstream signaling pathways by activating kinases, including FAK and Src [38]. Cell–cell junctions, including tight junctions and gap junctions, also participate in mechanical signal transduction [39]. Additionally, mechanical properties of the ECM, such as stiffness and viscoelasticity, influence nuclear transcription factor activity by modulating cytoskeletal tension. For instance, the mechanosensitive transcriptional co‑activators, YAP/TAZ, exhibits nuclear localization in a cytoskeletal tension-dependent manner, thereby regulating cell proliferation and differentiation [40]. These findings indicate that cells integrate physical signals from their microenvironment through a multidimensional mechanosensing system.

### Mechanotransduction pathways

Mechanical signals regulate cellular functions by activating multiple signaling pathways, among which the Mitogen-Activated Protein Kinase (MAPK), Phosphoinositide 3-Kinase (PI3K)/Protein Kinase B (Akt), and Hippo-YAP pathways serve as core transduction systems. In the MAPK pathway, ERK, c-Jun N-Terminal Kinase (JNK), and p38 mediate distinct responses to mechanical stimuli. Actin bundles provide a platform for ERK activation under mechanical tension, thereby promoting cell proliferation [41, 42]. Conversely, cyclic stretching activates the JNK pathway, which regulates ECs rearrangement [43]. The PI3K/Akt pathway is crucial for mechanotransduction-mediated cell survival and metabolic regulation. For instance, mechanical stretching promotes osteogenic differentiation while inhibiting adipogenesis in bone marrow mesenchymal stem cells (BMSCs) through PI3K/Akt pathway activation [44]. YAP and TAZ are well-characterized mechanosensors that shuttle between the cytoplasm and nucleus in response to cytoskeletal tension [45, 46]. The Hippo-YAP pathway acts as a central transcriptional regulatory system for mechanical signals. When adipose-derived stem cells were encapsulated in gelatin methacryloyl (GelMA) hydrogels of varying stiffness, a soft matrix (≈8 kPa) promoted YAP nuclear localization, whereas a stiff matrix (≈30 kPa) inhibited YAP activity, thereby directing stem cell differentiation [47]. However, YAP activation is not exclusively linked to soft substrates. Mammary epithelial cells and mesenchymal stem cells (MSCs) cultured on stiff hydrogels (15–40 kPa) exhibited YAP/TAZ activity and nuclear localization comparable to cells grown on plastic. In contrast, cells cultured on soft matrices (0.7–1 kPa) showed suppressed YAP/TAZ activity to levels similar to those achieved by siRNA-mediated knockdown [48].

The AMPK pathway is a central energy sensor that regulates cellular metabolism in response to mechanical and metabolic stress [49, 50]. As a member of the nuclear receptor tissue homeostasis by regulating cellular metabolism and inflammatory responses. For instance, mechanical stretching activates the AMPK pathway to promote mitochondrial biogenesis and fatty acid oxidation, thereby improving cellular metabolism [51, 52]. Within inflammatory microenvironments, mechanical signals influence the balance between glycolysis and oxidative phosphorylation by modulating the PPARγ co activator-1α (PGC-1α)/Lactate Dehydrogenase A (LDHA) axis, consequently affecting bone remodeling [53]. Mechanical signals participate in immune responses by regulating inflammatory factor expression. For example, cyclic stretch can suppress Nuclear Factor kappa B (NF-κB) pathway activation, reducing the release of pro‑inflammatory cytokines, such as Interleukin-6 (IL‑6) and Tumor necrosis factor-alpha (TNF‑α) [54].

The coordinated action of these downstream signaling pathways enables mechanical signals to precisely regulate cell differentiation, metabolism, and inflammatory responses, thereby providing a molecular foundation for understanding the role of mechanical forces in physiological and pathological processes.

## The complex biology of PPARγ as a mechano–metabolic transducer in lipid homeostasis

Having established how cells sense mechanical signals, we now discuss the complex biology of PPARγ and its role as a mechano–metabolic transducer in lipid homeostasis. PPARγ has a central role in adipogenesis and insulin sensitization. However, PPARγ also functions as a mechano–metabolic transducer, capable of converting physical forces into lipid metabolism changes. Understanding this dual role requires first a close look at its structure, activation mechanisms, and canonical biological functions.

### Structure and activation mechanisms of PPARγ

**Fig. 1:**
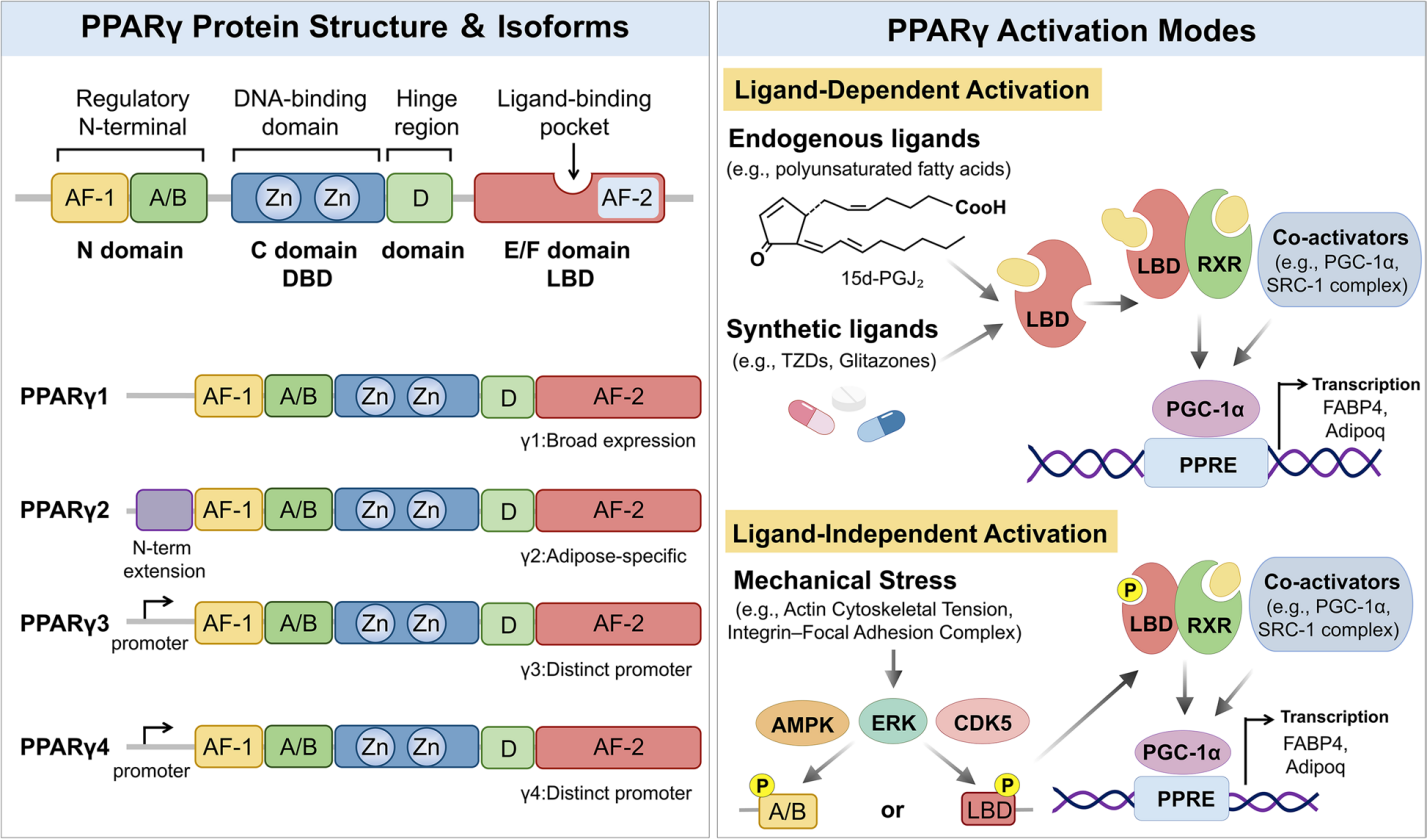
Structure, isoforms, and activation modes of PPARγ. The typical protein domain structure of PPARγ consists of a regulatory N‑terminal domain containing activation function‑1 (AF‑1; A/B domain), a DNA‑binding domain with zinc‑finger motifs (C domain/DBD), a hinge region (D domain), and a ligand‑binding domain containing activation function‑2 (AF‑2; E/F domain/LBD). Alternative splicing generates four isoforms: γ1 (ubiquitous), γ2 (adipose‑specific, with an N‑terminal extension), γ3, and γ4. PPARγ is activated through two main mechanisms. The first is ligand‑dependent activation. Endogenous (e.g., polyunsaturated fatty acids, 15d‑PGJ₂) or synthetic ligands (e.g., thiazolidinediones) bind the LBD, induce heterodimerization with RXR, recruit co‑activators (PGC‑1α, SRC‑1), and drive transcription of target genes (e.g., FABP4, Adipoq) via PPREs. The second mechanism is ligand‑independent activation. Mechanical cues (cytoskeletal tension, integrin‑focal adhesions) activate upstream kinases (AMPK, ERK, CDK5) that phosphorylate PPARγ, altering co‑factor interactions and transcriptional activity. Abbreviations: PPARγ, Peroxisome Proliferator-Activated Receptor Gamma; AF-1, activation function-1; AF-2, activation function-2; DBD, DNA-binding domain; LBD, ligand-binding domain; 15d-PGJ₂, 15-deoxy-delta(12, 14)-prostaglandin J(2); RXR, Retinoid X Receptor; PGC-1α, PPARγ Co-activator-1α; SRC-1, Steroid Receptor Co-activator-1; PPREs, peroxisome proliferator response elements; FABP4, Fatty Acid Binding Protein 4; Adipoq, Adiponectin; AMPK, AMP-Activated Protein Kinase; ERK, Extracellular Signal-Regulated Kinase; CDK5, Cyclin-Dependent Kinase 5

PPARγ is a member of the nuclear receptor superfamily and functions as a ligand-activated transcription factor [55, 56]. As a member of the nuclear receptor superfamily, PPARγ exhibits a typical modular structure. Similar to other PPAR subtypes, PPARγ comprises a highly conserved N-terminal DNA-binding and C-terminal ligand-binding domain. Its ligand-binding pocket is large, Y-shaped, and features three extended arm-like sub-pockets. This unique conformation enables the accommodation of and response to a diverse array of endogenous and synthetic ligands [57–61]. In humans, the *PPARG* gene produces four major mRNA isoforms, PPARγ1, γ2, γ3, and γ4, via distinct promoters and alternative splicing. PPARγ1 and PPARγ3 encode an identical protein and are widely expressed across multiple tissues. In contrast, PPARγ2 contains an additional 5′ exon encoding an extra 30 N-terminal amino acids; it is expressed predominantly in adipose tissue and plays a crucial role in adipogenesis and insulin sensitization [62]. However, the expression and distribution of PPARγ4 remains unclear. This isoform-specific expression pattern provides the structural basis for diverse physiological functions of PPARγ (Fig. 1).

PPARγ activation depends on ligand binding, with ligands categorized as endogenous or synthetic. Endogenous ligands, primarily derived from lipid metabolism, include polyunsaturated fatty acids, oxidized fatty acids (e.g., 9-Hydroxyoctadecadienoic Acid [9-HODE] and 13-HODE), and derivatives, such as 15-deoxy-delta(12, 14)-prostaglandin J(2) (15d-PGJ₂), thereby directly linking nutritional status to transcriptional regulation [63–66]. Clinically, synthetic ligands are more prominent, most notably, the thiazolidinedione (TZD) class of drugs, including rosiglitazone and pioglitazone. These potent PPARγ agonists are widely used to enhance insulin sensitivity in T2D [67–70]. However, due to adverse effects associated with TZDs, such as weight gain, edema, and increased fracture risk, a new generation of selective PPARγ modulators (Selective PPARγ Modulators [SPPARγMs]; e.g., INT131) has been developed. These compounds selectively recruit specific co-activator complexes, maintaining glycemic efficacy while mitigating the classic TZD-related side effects [71–73].

In addition to ligand-dependent activation, PPARγ can also be activated through ligand-independent mechanisms. Mechanical cues, such as cytoskeletal tension and integrin-focal adhesion complexes, activate upstream kinases (AMPK, ERK, cyclin-dependent kinase 5 [CDK5]), which phosphorylate PPARγ at specific residues, thereby altering its interactions with co-activators or -repressors and modulating its transcriptional activity [74, 75]. This ligand-independent mechano-activation reveals that PPARγ not only responds to biochemical signals but also senses and transduces physical information from the microenvironment. YAP/TAZ, which are well-established mechanosensors, interact directly with PPARγ and regulate its transcriptional activity in response to mechanical cues, such as matrix stiffness and stretch [76–79]. Consequently, PPARγ is increasingly recognized not merely as a classical nuclear receptor for lipid metabolism but as a “mechano–metabolic transducer” that integrates upstream mechanical signals to orchestrate lipid metabolic programs.

At the molecular level, ligand binding induces a conformational change in PPARγ, enabling it to heterodimerize with retinoid X receptor (RXR). The complex then binds to Peroxisome-proliferator response elements (PPREs) in target-gene promoters, recruiting co-activators, such as PGC-1α, to assemble an active transcription initiation complex [78–80]. PGC-1α does not bind DNA directly but acts as a potent transcriptional co-activator recruited to the PPARγ-RXR heterodimer. By recruiting chromatin-remodeling complexes, including histone acetyltransferases, it markedly amplifies PPARγ-driven transcription [78, 81–85]. The synergy between PPARγ and PGC-1α is critical for energy metabolism. In brown adipose tissue and skeletal muscle, this axis co-regulates mitochondrial biogenesis, fatty-acid β-oxidation, and thermogenic programs (e.g., uncoupling protein 1 [UCP1] expression), thereby promoting energy expenditure [86]. This interaction is modulated by metabolic status: during energy deprivation (e.g., fasting), Sirtuin 1 (Sirt1) is activated and deacetylates PGC-1α, enhancing its interaction with PPARγ and its co-activator capacity. This drives adaptive responses, such as fatty-acid oxidation and gluconeogenesis. Thus, the PPARγ-PGC-1α axis functions as a core molecular bridge that coordinates systemic energy homeostasis by linking nutrient sensing with transcriptional programs.

PPARγ activity is subject to multi-layered, precise regulation. Transcriptionally, the tissue-specific expression profile and cistrome are shaped by distinct chromatin environments and pioneer transcription factors. In macrophages, for example, PU.1 remodels chromatin accessibility to facilitate PPARγ binding. In white adipocytes, different factors, such as CCAAT/enhancer binding protein (C/EBP) family members, direct PPARγ to adipogenic enhancers [87]. Post-transcriptionally, covalent modifications fine-tune PPARγ stability and transcriptional activity. Phosphorylation is a key regulatory mechanism: CDK5-mediated phosphorylation of PPARγ (e.g., at Ser273) shifts its target gene repertoire toward insulin-resistance-associated genes. Inhibiting CDK5 or using non-agonistic ligands blocks its phosphorylation, thereby improving insulin sensitivity [88]. SUMOylation is central to PPARγ-mediated transcriptional repression. Ligand-activated PPARγ can be SUMOylated and recruited to inflammatory-gene promoters (e.g., NF-κB targets), where it sustains repression by preventing corepressor-complex clearance, a mechanism termed transrepression [89]. Furthermore, the acetylation-deacetylation balance also modulates PPARγ function. For instance, the deacetylase, Sirt1, can indirectly influence PPARγ transcriptional activity by deacetylating PGC-1α [90].

### Biological functions mediated by PPARγ

PPARγ is widely recognized as the master transcriptional regulator of adipocyte differentiation [91, 92]. PPARγ is involved in diverse cellular physiological functions, playing important roles in regulating adipocyte differentiation, lipid and carbohydrate metabolism, inflammatory responses, and cell proliferation and differentiation [93–95]. PPARγ acts as a key transcription factor in adipocyte differentiation. It synergizes with C/EBP family proteins to activate adipocyte-specific genes, such as *fatty acid binding protein 4 (FABP4)* and *ADIPOQ*, thereby promoting the differentiation of preadipocytes into mature adipocytes [96, 97]. PPARγ also participates in glucose metabolism by enhancing insulin sensitivity. It upregulates the expression of the glucose transporter type 4 (GLUT-4), increases glucose uptake and utilization, and reduces blood glucose levels. This function establishes PPARγ as a major therapeutic target in T2D [45, 98, 99]. As a central regulator of lipid metabolism, PPARγ maintains the balance of the lipid metabolic network by modulating the expression of related genes. During lipogenesis, PPARγ activation promotes the expression of genes, such as *fatty acid synthase* (*FAS*) and *acetyl-CoA carboxylase* (*ACC*), thereby increasing triglyceride synthesis and storage [100]. Concurrently, PPARγ activation can promote the expression of genes involved in fatty acid oxidation, such as *carnitine palmitoyltransferase 1 A (CPT1A)*, enhancing energy expenditure [101]. In cholesterol metabolism, PPARγ promotes reverse cholesterol transport by regulating the expression of genes, such as *ATP-binding cassette transporter A1 (ABCA1) and ATP-binding cassette transporter G1 (ABCG1)*, thereby lowering plasma cholesterol levels [102]. The PPARγ-liver X receptor alpha (LXRα)-ABCA1 axis is a well-established pathway that mediates reverse cholesterol transport [55, 103]. For example, polymethoxyflavones promote cholesterol efflux in macrophages and inhibit foam cell formation by activating the PPARγ/LXRα pathway [102]. The regulatory role of PPARγ in lipid metabolism is also evident in its modulation of adipocyte phenotype. PPARγ activation promotes the “browning” of white adipocytes, increasing UCP1 expression and enhancing thermogenesis [101]. Furthermore, PPARγ influences systemic metabolism by regulating adipokine secretion. Adiponectin, a target gene of PPARγ, improves insulin sensitivity and suppresses inflammation [104, 105]. These findings demonstrate that PPARγ maintains lipid metabolic homeostasis through multidimensional regulatory mechanisms.

In addition to its roles in lipid metabolism, PPARγ plays a pivotal role in cardiovascular health, particularly in modulating atherosclerosis and maintaining vascular homeostasis. In atherosclerotic lesions, the PPARα/γ axis attenuates inflammatory responses in vascular cells [106]. Moreover, in various cancer types, ligand-mediated PPARγ activation can inhibit cell proliferation, establishing it as a potential therapeutic target. PPARγ is recognized as a tumor suppressor; activation of the PPARγ/RXRα signaling pathway inhibits tumor progression in colon, breast, prostate, and bladder cancers [107]. Furthermore, PPARγ agonists show efficacy in experimental models of several neurodegenerative and brain injury conditions, including Parkinson’s disease, amyotrophic lateral sclerosis, Alzheimer’s disease, and traumatic brain injury [108, 109]. In ocular diseases, PPARγ ligands exhibit therapeutic potential, particularly in inhibiting corneal neovascularization, reducing fibrosis following alkali burns, inducing apoptosis in human pterygium fibroblasts, and ameliorating conditions, such as age-related macular degeneration, diabetic retinopathy, keratitis, and optic neuropathy [110].

**Fig. 2:**
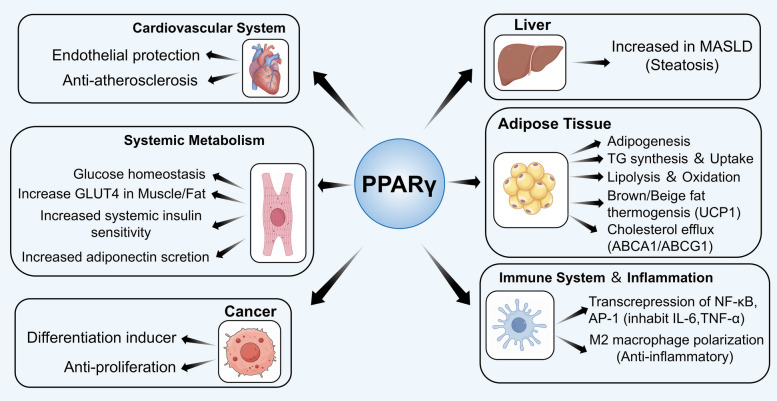
Biological function network of PPARγ. As a core transcription factor of lipid metabolism, PPARγ exerts broad regulatory effects across multiple tissues. In the cardiovascular system, PPARγ shows endothelial‑protective and anti‑atherosclerotic functions by suppressing inflammatory responses and maintaining vascular homeostasis. In the liver, its expression is increased in MASLD, where it promotes hepatocellular lipid accumulation and steatosis. At the level of systemic metabolism, PPARγ enhances insulin sensitivity and maintains glucose homeostasis by increasing adiponectin secretion and upregulating the expression of the glucose transporter GLUT-4 in muscle and adipose tissues. In adipose tissue, PPARγ promotes adipogenesis as well as TG synthesis and uptake, regulates lipolysis and fatty‑acid oxidation, and induces thermogenesis in brown and beige adipose tissue through upregulation of UCP1. At the same time, it facilitates cholesterol efflux by increasing the expression of ABCA1 and ABCG1. In tumor cells, PPARγ can induce cell differentiation and inhibit excessive proliferation, reflecting its potential anti‑tumor activity. In addition, within the immune system, PPARγ suppresses NF‑κB‑ and AP‑1‑mediated inflammatory signaling by reducing the expression of IL‑6 and TNF‑α, and promotes macrophage polarization toward the M2 phenotype, thereby exerting anti‑inflammatory effects. Overall, by coordinating lipid metabolism, glucose homeostasis, inflammatory regulation, and cell differentiation, PPARγ functions as a central hub in the control of systemic energy metabolism and tissue homeostasis. Abbreviations: PPARγ, peroxisome proliferator-activated receptor gamma; MASLD, metabolic dysfunction-associated steatotic liver disease; GLUT-4, glucose transporter type 4; TG, triglyceride; UCP1, uncoupling protein 1; ABCA1, ATP-binding cassette transporter A1; ABCG1, ATP-binding cassette transporter G1; NF-κB, Nuclear Factor kappa B; AP-1, activator protein-1; IL-6, Interleukin-6; TNF-α, Tumor necrosis factor-alpha

The regulatory effects of PPARγ are tissue-specific. In the liver, its activation can promote lipogenesis and hepatic steatosis, whereas in the adipose tissue, it improves metabolic parameters partly by increasing adiponectin secretion [111, 112]. Furthermore, PPARγ expression and activity are dynamically regulated by various factors. For example, mechanical signals can influence adipocyte differentiation by modulating PPARγ expression, while inflammatory cytokines, such as TNF-α, can promote insulin resistance by inhibiting PPARγ activity [113, 114].Of note, the PPARγ regulatory network extensively cross-talks with other signaling pathways. The AMPK pathway promotes mitochondrial biogenesis and fatty acid oxidation, partly through PGC-1α axis activation [115]. This interconnectivity is also observed in the context of neurodegenerative diseases, where AMPK, Sirt1, and PGC-1α form an integrated signaling network that modulates mitochondrial function and cellular energy homeostasis [116].These observations indicate that as a core regulator of lipid metabolism, the precise control of PPARγ function depends on the coordinated action of complex signaling networks and the tissue microenvironment (Fig. 2). Collectively, PPARγ is a central lipid metabolism regulator in response to biochemical signals as well as a key mechano‑metabolic transducer that converts physical forces into metabolic adaptations.

## Molecular mechanisms of PPARγ in transducing mechanical signals to regulate lipid metabolism

Mechanical signals such as matrix stiffness, cyclic stretch, and fluid shear stress do not act directly on metabolic genes; they have to be relayed through specific signaling cascades. PPARγ sits at the receiving end of several such cascades, where its expression, activity, and post-translational modifications are finely tuned. This section discusses the molecular pathways that bridge mechanical cues to PPARγ-mediated lipid regulation.

### Regulation of PPARγ expression by mechanical signals

Matrix stiffness is a critical physical cue that directs mesenchymal stem cell lineage specification [117, 118]. PPARγ is a key regulator of adipogenesis and energy metabolism. Although PPARγ is not a direct mechanosensor, its expression and activity are precisely regulated by mechanical signals, including matrix stiffness, tensile strain, fluid shear stress, and hydrostatic pressure. This establishes PPARγ as a critical downstream effector in mechanotransduction pathways that control cell differentiation and tissue homeostasis [6, 16, 47, 119, 120].

#### Matrix stiffness and the three-dimensional (3D) microenvironment regulate PPARγ activity

The physical properties of ECM, particularly its stiffness, influence the lineage commitment of MSCs. Soft matrices promote adipogenic differentiation via PPARγ upregulation, whereas stiff matrices favor osteogenesis through Runt-related transcription factor 2 (Runx2) activation [117, 121]. Reduced mechanical rigidity in polyurethane-ester-ether scaffolds with gradient stiffness significantly increased adipose tissue formation, indicating that mechanical signals alone can drive adipogenic differentiation via PPARγ activation [122, 123]. However, this relationship is microenvironment-dependent. While soft substrates promote PPARγ in conventional two-dimensional (2D) culture, high stiffness can paradoxically enhance PPARγ expression under confined 3D conditions, highlighting the complexity introduced by dimensionality and spatial constraint [15, 47].

#### Tensile strain and cyclic stretching suppress PPARγ to promote osteogenesis

Mechanical stretching is a potent regulator of PPARγ expression and activity, predominantly by downregulating PPARγ. In BMSCs, physiological tensile strain inhibits miR-140-5p expression by upregulating lncRNA-MEG3 expression. This establishes a lncRNA-MEG3-miR-140-5p-osteogenic transcription factor axis that enhances osteogenesis and suppresses adipogenesis [124]. In the pluripotent mesenchymal precursor line C3H10T1/2, cyclic mechanical stretching significantly reduces PPARγ2 protein levels and antagonizes its ligand-binding domain activity. Consequently, it blocks rosiglitazone-induced adipogenic programs, an effect maintained even under pro-adipogenic conditions, such as high insulin levels [6, 21]. Collectively, these studies demonstrate that mechanical stretch-mediated suppression of PPARγ is a central molecular mechanism underlying the anti-adipogenic, pro-osteogenic effect of mechanical loading.

#### Fluid shear stress and hemodynamic forces regulate vascular PPARγ

In ECs, aberrant hemodynamic signals, such as portal hypertension-induced oscillatory shear flow or mechanical stress, suppress PPARγ expression and activity via multiple pathways [16]. OSS is a pro-inflammatory mechanical stimulus that promotes endothelial dysfunction [125, 126]. In human umbilical vein endothelial cells (HUVECs), non-physiological impinging flow (but not laminar flow) rapidly activates PKCα, leading to phosphorylation of the ERK/MAPK pathway. This cascade downregulates PPARγ transcription while promoting nuclear translocation of NF-κB and upregulating matrix metalloproteinase 2 (MMP2), which are key events in endothelial dysfunction and aneurysm development. Sustained mechanical stretching, mimicking portal hypertension, reduces endothelial PPARγ levels through two parallel mechanisms: (i) transcriptional inhibition via blockade of the PI3K/AKT/cyclic-AMP response element-binding protein (CREB) axis, and (ii) post-translational degradation via the E3 ubiquitin ligase NEDD4, which is upregulated through the Reactive oxygen species (ROS)/NF-κB pathway and mediates K48-linked ubiquitination and subsequent proteasomal degradation of PPARγ [16]. The resulting PPARγ inactivation relieves its repression of endothelial-to-mesenchymal transition (EndMT), thereby driving vascular fibrosis.

#### High-energy mechanical stimulation suppresses adipogenesis via PPARγ

Non-invasive mechanical interventions, such as low-intensity shockwave therapy, exert anti-adipogenic effects partly by downregulating PPARγ. In 3T3-L1 cells and primary preadipocyte models, physiological low-energy shockwaves inhibit PPARγ-driven adipogenic differentiation through two mechanisms: (i) reducing intracellular cyclic adenosine monophosphate (cAMP) levels to directly inhibit cAMP/protein kinase A (PKA) pathway activation and pro-adipogenic transcription factors (e.g., C/EBPα); and (ii) enhancing β-catenin stability by attenuating its phosphorylation-dependent degradation, allowing nuclear β-catenin to competitively inhibit PPARγ transcriptional activity. Mechanistically, exogenous supplementation with a cAMP analog (e.g., dbcAMP) completely reverses the anti-adipogenic effect of shockwaves, establishing the cAMP/Wnt/β-catenin axis upstream of PPARγ as a core node transducing mechanical signals into metabolic phenotypes [127]. These studies delineated a regulatory pathway from mechanical stimuli to transcription factors to metabolic outcomes, providing a mechanistic rationale for non-invasive mechanical interventions as potential therapies for obesity and metabolic disorders.

### Integrated signaling pathways linking mechanical forces to PPARγ

**Fig. 3:**
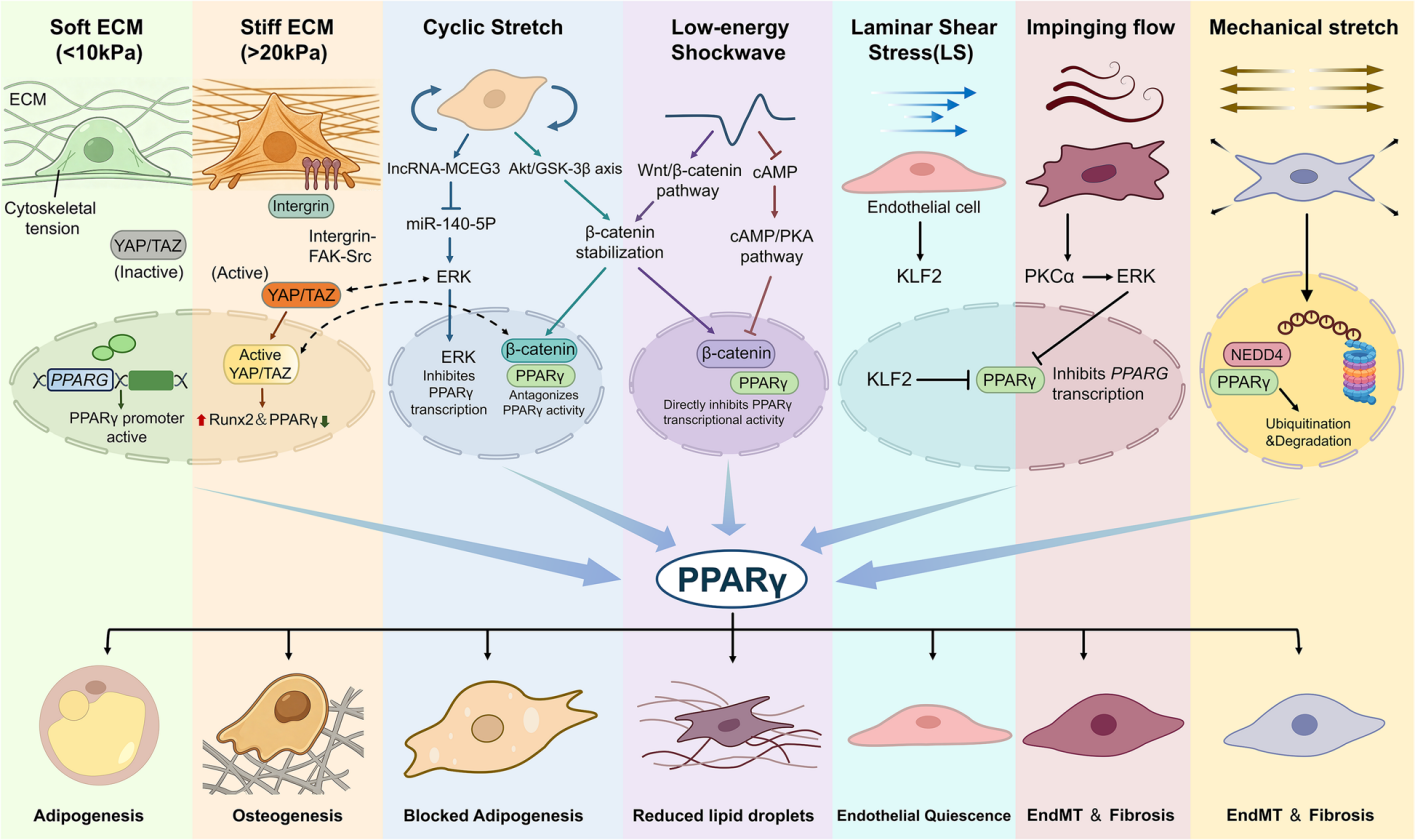
Schematic diagram of molecular pathways by which mechanical signals regulate PPARγ. Distinct mechanical cues, including matrix stiffness, cyclic stretch, fluid shear stress, and low energy shockwaves, activate specific upstream signaling cascades that converge on PPARγ to regulate its expression and transcriptional activity, thereby determining cell fate and metabolic phenotype. Regarding matrix stiffness, a soft matrix (< 10 kPa) reduces cytoskeletal tension and retains YAP/TAZ in the cytoplasm, relieving their repression of the PPARγ promoter and promoting adipogenesis; in contrast, a stiff matrix (> 20 kPa) activates the integrin-FAK-Src axis, leading to YAP/TAZ nuclear translocation, which upregulates Runx2 and directly suppresses PPARγ expression, driving osteogenesis. Cyclic stretching negatively regulates PPARγ through two parallel pathways: it upregulates lncRNA-MEG3, which inhibits miR-140-5p and activates ERK signaling to downregulate PPARγ2 expression, and it activates the Akt/GSK- 3β axis to stabilize β-catenin, which translocates to the nucleus and antagonizes PPARγ transcriptional activity; both pathways block adipogenesis. Low energy shockwave stimulation reduces intracellular cAMP levels, suppressing the cAMP/PKA pathway and activating the Wnt/β-catenin pathway; stabilized β-catenin translocates into the nucleus and directly inhibits PPARγ transcriptional activity, reducing lipid droplet formation and suppressing adipogenesis. Under fluid shear stress, laminar shear stress induces KLF2 expression and suppresses PPARγ transcription, maintaining endothelial quiescence, whereas impinging flow and mechanical stretch activate the PKCα-ERK pathway to inhibit PPARγ transcription and promote NEDD4-mediated PPARγ ubiquitination, leading to EndMT and fibrosis. Overall, distinct mechanical cues regulate PPARγ function through specific signaling networks at both transcriptional and post-translational levels, forming the molecular basis of the “mechanical signal–PPARγ–cell fate/lipid metabolism” regulatory axis. Notably, these pathways are not entirely independent; for example, ERK activation downstream of cyclic stretch has been shown to modulate YAP/TAZ activity, and β-catenin can interact with YAP/TAZ in the nucleus, forming a broader interconnected network that converges on PPARγ regulation. Abbreviations: PPARγ, peroxisome proliferator-activated receptor gamma; YAP, Yes-associated protein; TAZ, transcriptional coactivator with PDZ-binding motif; Runx2, Runt-related transcription factor 2; FAK, focal adhesion kinase; Src, proto-oncogene tyrosine-protein kinase Src; lncRNA-MEG3, long non-coding RNA maternally expressed gene 3; miR-140-5p, microRNA-140-5p; Akt, protein kinase B; GSK-3β, Glycogen synthase kinase-3 beta; cAMP, cyclic adenosine monophosphate; PKA, protein kinase A; KLF2, Krüppel-like factor 2; PKCα, protein kinase C alpha; NEDD4, neural precursor cell expressed developmentally down-regulated protein 4; EndMT, endothelial-to-mesenchymal transition

Importantly, PPARγ is not a direct mechanosensor; it does not bind to or directly respond to physical forces. Instead, PPARγ serves as a central downstream integrator and transcriptional transducer. Mechanical signals (e.g., stiffness, shear stress, and stretch) are first sensed by specialized mechanoreceptors (Piezo1 and integrins) and transduced through cascades (YAP/TAZ, ERK, and NEDD4) that ultimately converge on PPARγ, which then orchestrate lipid metabolic gene expression. Thus, PPARγ transduces mechanical information into a metabolic phenotype at the transcriptional level. Mechanical signals are transduced through several conserved signaling cascades, including MAPK, PI3K/AKT, and Hippo-YAP pathways [128, 129]. As a core responder to changes in the mechanical microenvironment, PPARγ expression and activity are regulated by complex signaling networks. The key regulatory mechanisms are summarized below (Fig. 3).

#### Mechanical microenvironment regulates PPARγ activity via the YAP/TAZ-Hippo pathway

YAP and TAZ, effector molecules of the Hippo signaling pathway, have been identified as nuclear relays of mechanical signals exerted by ECM rigidity and cell shape, serving as sensors and mediators of mechanical cues instructed by the cellular microenvironment [45]. In 2D culture, high matrix stiffness promotes cell spreading, activates YAP/TAZ, and thereby suppresses PPARγ transcriptional activity, inhibiting preadipocyte differentiation and promoting osteogenesis [14, 15]. The conserved negative regulatory role of YAP/TAZ in adipocyte differentiation is demonstrated by TAZ-mediated PPARγ transcriptional activity suppression and YAP1 knockdown-induced *PPARG* upregulation [130, 131]. It has been reported that TAZ suppresses PPARγ transcriptional activity by directly binding to the PPARγ PPXY motif through its WW domain, and this interaction is enhanced by ERK-mediated phosphorylation on PPARγ Ser112. This provides a mechanism for the negative regulation of adipogenesis under mechanical load [132]. This finding further supports the critical involvement of this mechanosensitive pathway in MSCs lineage commitment; cyclic mechanical stretching upregulates Runx2 and downregulated PPARγ2, directing cells toward an osteogenic fate [21, 133]. However, this regulatory relationship exhibits considerable complexity in 3D microenvironments. In confined 3D structures, even soft substrates may suppress PPARγ expression due to cell volume compression, whereas certain high-stiffness 3D scaffolds can favor PPARγ upregulation [47], highlighting the strong microenvironment-dependency of PPARγ mechanical regulation. Furthermore, YAP/TAZ function is stage- and cell-type-specific. For example, ubiquitous YAP1 overexpression from the zygotic stage leads to TAZ downregulation in adipose stem cells, which relieves PPARγ suppression and promotes adipocyte differentiation, ultimately inducing an obesity-like phenotype in mice [134].

Notably, the relationship between matrix stiffness and YAP/TAZ activity is context-dependent and differs between 2 and 3D culture systems [45, 47, 135]. Given the above findings on how soft versus stiff matrices regulate YAP/TAZ expression, contradictory results have been reported: some studies show that soft matrices promote YAP nuclear localization [47], whereas others indicate that stiff matrices enhance YAP/TAZ activity and soft matrices suppress it [14]. Similarly, in 2D culture, soft substrates promote PPARγ expression and adipogenesis by reducing YAP/TAZ nuclear translocation, thereby relieving transcriptional repression of the PPARγ promoter [15], whereas in confined 3D environments, high stiffness paradoxically enhances PPARγ expression due to cell volume compression and reduced YAP/TAZ activity [47]. We propose that these discrepancies are not true contradictions but rather reflect the context-dependent nature of mechanotransduction. First, culture dimensionality fundamentally alters mechanosensing.In 2D, stiff matrices increase cytoskeletal tension through the integrin–FAK–Ras homolog family member A (RhoA)/Rho-associated protein kinase (ROCK) axis, driving YAP/TAZ nuclear translocation [136], whereas soft matrices reduce tension and retain YAP/TAZ in the cytoplasm. In 3D, cells are surrounded by matrix and experience volumetric compression; even in soft 3D hydrogels, cells actively remodel their surroundings, generating local traction forces that can activate YAP/TAZ and override the effect of low global stiffness [47]. This explains why a “soft” matrix can produce opposite YAP/TAZ outcomes in 2D versus 3D. Second, cell type, stiffness range, and matrix composition play critical roles. YAP/TAZ mechanosensitivity thresholds vary among cell types (e.g., MSCs versus epithelial cells); a “soft” matrix in one study (e.g., 8 kPa) may be considered “stiff” in another (e.g., 0.7 kPa) [14, 45, 48, 137]; different matrix materials (GelMA, collagen, polyacrylamide) engage distinct integrin repertoires, thereby altering downstream signaling [138, 139]. Third, YAP/TAZ and PPARγ have bidirectional, context-dependent interactions. On stiff 2D matrices, activated YAP/TAZ represses the PPARγ promoter via Transcriptional Enhancer Associate Domain (TEAD)-dependent epigenetic mechanisms, blocking adipogenesis and promoting osteogenesis [140]. However, in 3D confined environments, high stiffness can paradoxically upregulate PPARγ by suppressing YAP/TAZ nuclear localization due to cell volume compression [47], indicating that the YAP/TAZ-PPARγ relationship is not a linear switch but a tunable sensor influenced by dimensionality and confinement. Fourth, matrix stiffness and viscoelasticity activate YAP/TAZ, which in turn influences cellular metabolism (e.g., glycolysis and glutamine dependence) and ECM remodeling in fibrosis and cancer [14, 137, 141]. Therefore, observations from one experimental system may not directly apply to another. In summary, the apparent contradictions in the literature underscore the importance of considering dimensionality, cell type, stiffness range, matrix composition, confinement, culture duration, and differentiation stage when interpreting mechano-based PPARγ regulation.

#### Dynamic mechanical loads regulate PPARγ via specific pathways

In addition to substrate mechanics, dynamic mechanical loads considerably influence PPARγ. Dynamic mechanical loading, including tensile strain and cyclic stretch, activates distinct mechanotransduction pathways compared to static stiffness [142, 143]. In bone tissue engineering, appropriate tensile strain upregulates lncRNA-MEG3, inhibits miR-140-5p, and promotes osteogenic differentiation in BMSCs, concomitant with downregulation of adipogenic factors, including PPARγ and SRY-box transcription factor 9 (SOX9) [124, 144]. Similarly, in tendon stem/progenitor cells, high-intensity mechanical load activates mammalian Target of Rapamycin (mTOR) signaling, increasing the expression of non-tendon lineage genes, such as *PPARG*, *SOX9*, and *Runx2*, which drives aberrant differentiation and contributes to tendinopathy [145]. Rapamycin-mediated inhibition of mTOR alleviates these changes, suggesting that the mTOR–PPARγ axis is a key pathway in mechanically triggered pathological remodeling. In the cardiovascular system, mechanical stress regulates PPARγ through distinct mechanisms. Under conditions mimicking portal hypertension, sustained stretching in ECs blocks the PI3K/AKT/CREB axis and enhances NEDD4-mediated PPARγ ubiquitination and degradation, ultimately triggering EndMT [16]. At arterial bifurcations under impinging flow, PKCα activation leads to phosphorylation of ERK and JNK pathways, inhibiting PPARγ expression, and promoting NF-κB and MMP2 upregulation, events that exacerbate vascular injury and contribute to intracranial aneurysm development [17]. Of note, PPARγ agonists, such as rosiglitazone, can reverse stretch-induced EndMT in vitro, indicating their potential therapeutic role in mechanical stress-related vascular pathologies [16].

### Regulatory roles of mechanical forces and PPARγ in lipid metabolism

As the master transcriptional regulator of adipogenesis, PPARγ governs preadipocyte fate and centrally regulates energy storage, insulin sensitivity, and inflammatory responses in mature adipocytes [96, 97, 104, 105]. Mechanical microenvironmental changes directly participate in adipose tissue metabolic remodeling by modulating PPARγ activity, thereby shaping lipid metabolic phenotypes in physiology and disease.

In adipose tissue homeostasis, appropriate mechanical tension helps restrain excessive lipid accumulation via low-intensity shockwave therapy [127]. This mechanism suggests that pulsed mechanical force could serve as a non-invasive intervention for regulating localized fat deposition. In thyroid eye disease, elevated pressure in the orbital tissue activates the mechanoreceptor, Piezo1. The Piezo1 agonist, Yoda1, suppresses key adipogenic factors, including PPARγ, C/EBPα, and FABP4, reducing the adipogenic conversion of orbital fibroblasts [113], indicating that mechanical signals can negatively regulate local fat deposition via the Piezo1-PPARγ axis.

Conversely, under the pathological conditions of chronic static loading or increased tissue stiffness, mechanical signals may promote lipid metabolism [134]. In 3D scaffold systems, reducing matrix stiffness significantly enhances adipose tissue regeneration, with a pro-adipogenic effect comparable to PPARγ agonist treatment [122]. This “soft-substrate preference” arises from reduced nuclear translocation of YAP/TAZ in mechanically permissive environments, relieving their PPARγ promoter repression and enabling adipogenic programming [130, 131]. Notably, in confined 3D cultures, high stiffness may indirectly suppress PPARγ through spatial restriction and cell compression [47], underscoring the dimension-dependency and microenvironment-specificity of mechanical regulation.

Mechanical forces can also modulate PPARγ activity via post-translational modifications [74, 75]. In a model of diabetic myocardial fibrosis, a high-glucose environment inhibits AMPK activity, reduces Enhancer of Zeste Homolog 2 (EZH2) phosphorylation, and transcriptionally suppresses PPARγ. This promotes fibroblast-to-myofibroblast transition [146]. Similarly, in hepatic stellate cells, GLIS family zinc finger 2 (GLIS2) competitively binds to histone deacetylase HDAC3, preventing PPARγ deacetylation and thereby maintaining PPARγ in an active state to inhibit cell activation and fibrosis linked to lipid metabolic imbalance [147]. Therefore, mechanical stress not only affects PPARγ expression but also fine-tunes its function through epigenetic and protein-modification mechanisms, thereby influencing cellular energy allocation.

At the systemic level, mechanosensing participates in the metabolic crosstalk between the adipose tissue and other organs. Medium-chain triglycerides, for example, upregulate PPARγ by activating AMPK signaling, promoting brown adipose tissue activation and browning, and enhancing energy expenditure [148]. Although primarily driven by biochemical signals, the accompanying tissue expansion and altered microenvironmental mechanics may further amplify the metabolic effects of PPARγ, establishing a “biochemical–mechanical” positive-feedback loop.

In summary, the interplay between mechanical forces and PPARγ in lipid metabolism is bidirectional, dynamic, and highly context-dependent. PPARγ acts as a key effector downstream of mechanical signals, with its activity precisely tuned by the cellular mechanical state [15, 45]. The PPARγ functional output influences tissue structural mechanics, forming an integrated mechano-metabolic network. It is worth noting that the regulatory mechanisms described above are not isolated linear pathways; under pathological conditions, they can converge into a self‑reinforcing vicious cycle. This cycle is triggered by aberrant mechanical signals, such as chronically increased matrix stiffness or pathological shear stress [16, 17, 47]. These signals suppress PPARγ expression and activity through pathways including YAP/TAZ and NEDD4-mediated ubiquitination. The resulting PPARγ dysfunction leads to metabolic dysregulation—impaired adipogenesis, ectopic lipid accumulation, and insulin resistance—and promotes tissue remodeling, most notably fibrosis and ECM deposition [45, 122]. During this process, decreased PPARγ activity can upregulate pro-fibrotic factors such as TGF-β and CTGF, accelerating collagen deposition and further increasing tissue stiffness, which amplifies the initial aberrant mechanical signals. This establishes a positive feedback loop of “mechanical signal imbalance-metabolic dysregulation-tissue remodeling-worsening mechanical imbalance.” This cyclical framework indicates that PPARγ is not merely a passive responder but rather a critical node whose dysfunction actively drives disease progression. In‑depth investigation of the operational principles of this regulatory axis across different tissues and disease contexts, along with validation of the conservation of this vicious cycle in in vivo models, will not only help elucidate the fundamental mechanisms of lipid metabolism but also provide a solid theoretical foundation for developing metabolic intervention strategies based on physical stimuli.

In addition, the pathways summarized above (YAP/TAZ, PKCα-ERK, NEDD4, and cAMP/Wnt/β-catenin) have been validated by direct experimental approaches in specific cell types. However, several important questions remain: (i) whether these pathways operate independently or interact in a cell-type-specific manner; (ii) how 2D vs. 3D culture conditions affect the outcomes; (iii) whether the observed effects are reversible; and (iv) whether the magnitude and duration of mechanical stimuli used experimentally reflect physiological or pathophysiological conditions. Most studies have used supraphysiological levels of stiffness, stretch, or shear stress [16, 17, 145]; however, the relevance of subtle, chronic mechanical alterations in native tissues requires further investigation.

## Mechano‑PPARγ axis in physiological and pathological lipid homeostasis

Whether mechanical PPARγ regulation leads to health or disease depends heavily on the tissue context and the nature of the mechanical stimulus. In healthy tissues, appropriate mechanical forces help maintain metabolic balance; in disease, aberrant signals drive a vicious cycle of PPARγ dysfunction and tissue remodeling. This section examines how this axis operates in physiological settings, such as embryonic development and exercise, and how it becomes derailed in obesity, atherosclerosis, MASLD, and diabetes.

### Regulation under physiological conditions

In normal physiological conditions, mechanical forces are critical for maintaining tissue homeostasis and guiding cell differentiation. Mechanical forces are essential for maintaining tissue homeostasis and regulating stem cell differentiation [19, 149]. As a sensitive node for mechanical signals, PPARγ plays important roles in embryonic development, exercise adaptation, and vascular function.

#### Mechanical regulation of lipid metabolism via PPARγ in embryonic development

**Fig. 4:**
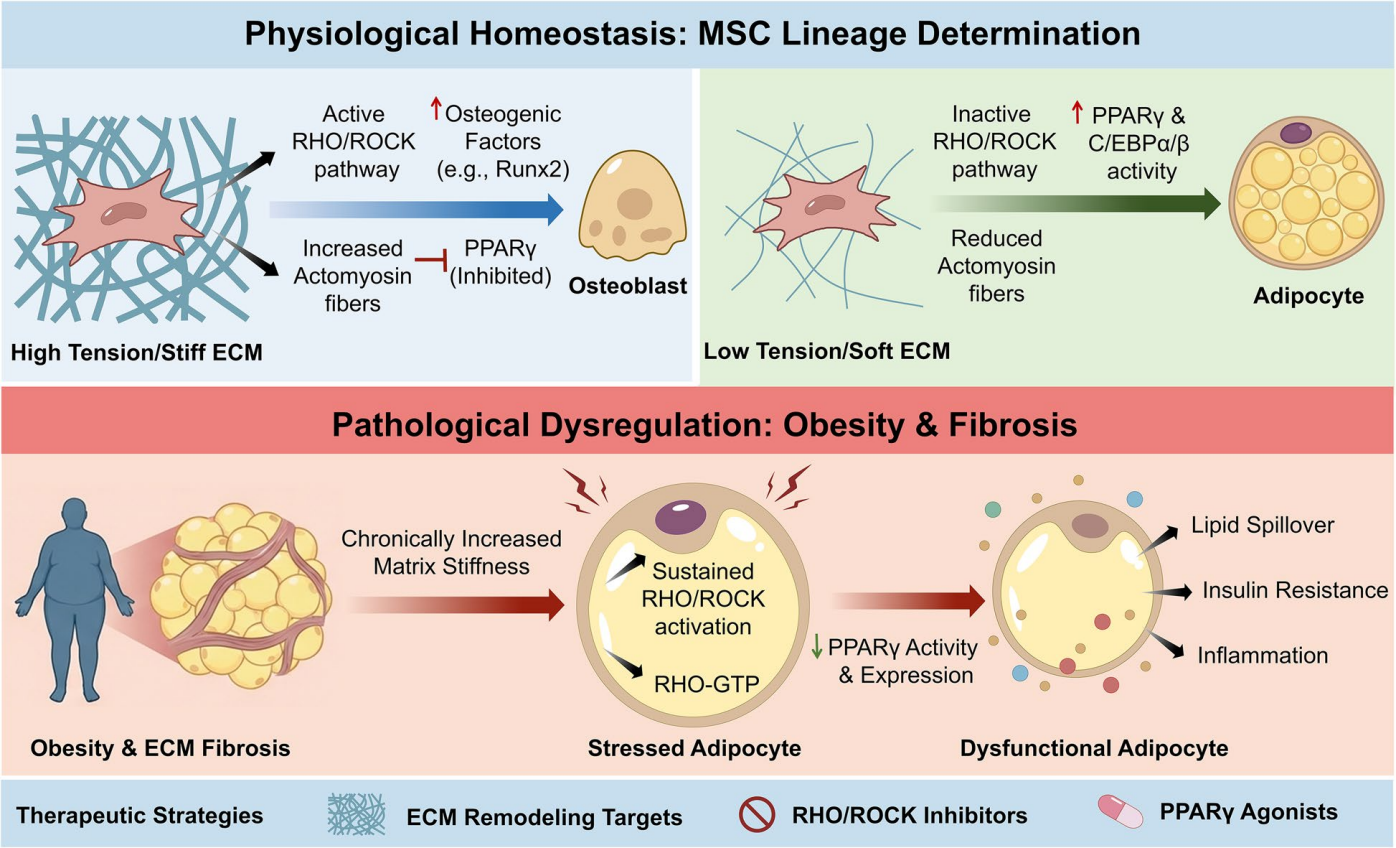
PPARγ‑mediated mechano‑metabolic regulation under physiological and pathological conditions. Under physiological conditions, cells sense distinct mechanical microenvironments and modulate PPARγ activity through the RHO/ROCK signaling pathway, thereby regulating MSCs fate determination and adipose tissue function. In high‑tension or stiff‑matrix environments (such as bone tissue), the RHO/ROCK pathway is activated, promoting myosin‑based stress fiber formation and upregulating osteogenic transcription factors (e.g., Runx2), while concomitantly suppressing PPARγ activity, thus driving MSCs differentiation toward the osteogenic lineage. In contrast, in low‑tension or soft‑matrix environments (such as healthy adipose tissue), the RHO/ROCK pathway remains inactive, cytoskeletal tension is reduced, and PPARγ together with its cooperative factors C/EBPα and C/EBPβ is fully activated. This activation induces the expression of adipogenic genes, promotes MSCs differentiation into mature adipocytes, and facilitates lipid storage. Under pathological conditions, chronic ECM fibrosis leads to a sustained increase in matrix stiffness, resulting in persistent activation of the RHO/ROCK pathway and elevated RHO‑GTP levels. This, in turn, suppresses PPARγ expression and activity, disrupts adipocyte metabolic and endocrine functions, and causes lipid spillover, insulin resistance, and chronic inflammation, ultimately leading to functional adipocyte dysfunction. Based on this mechano‑regulatory framework, potential therapeutic strategies include promoting ECM remodeling to restore tissue compliance, applying RHO/ROCK inhibitors to block aberrant mechanical signaling, or using PPARγ agonists to enhance PPARγ activity and thereby improve metabolic homeostasis. Abbreviations: PPARγ, peroxisome proliferator-activated receptor gamma; RHO, Ras homolog family member; ROCK, Rho-associated protein kinase; MSCs, mesenchymal stem cells; Runx2, Runt-related transcription factor 2; C/EBPα, CCAAT/enhancer-binding protein alpha; C/EBPβ, CCAAT/enhancer-binding protein beta; ECM, extracellular matrix; ECM, extracellular matrix

The mechanical microenvironment is crucial for determining MSCs lineage commitment. Mechanical loading suppresses PPARγ and promotes osteogenesis through the activation of Runx2 [143, 150]. In tissue-culture models, periodic mechanical loading significantly increases levels of the osteogenic transcription factor, Runx2, and decreases PPARγ2 expression [151, 152]. Therefore, during early embryonic development, dynamic forces generated by blood flow, cell proliferation, and tissue expansion may guide MSCs toward an osteogenic fate by suppressing PPARγ-driven adipogenesis, thereby ensuring proper skeletal formation. In this process, PPARγ acts as a “mechano-biochemical” signal converter, whose activity level directly determines whether a cell commits to an adipogenic or osteogenic lineage (Fig. 4).

#### Mechanical lipid metabolism regulation via PPARγ in exercise and loading

Exercise is a classic physiological mechanical stimulus and its benefits for lipid metabolism partly arise from direct modulation of PPARγ. Low-magnitude mechanical signals (LMMS) effectively prevent diet-induced obesity. In C57BL/6 J mice, a six-week LMMS intervention upregulated Runx2 by 72% and downregulated PPARγ by 27% in bone marrow MSCs, significantly inhibiting visceral adipose tissue formation [153, 154]. In vitro studies further show that applying cyclic stretching to MSCs in adipogenic medium suppresses PPARγ expression via activation of the ERK pathway, thereby blocking adipocyte differentiation [152, 155]. Mechanical strain also activates the AKT/GSK-3β/β-catenin axis, which stabilizes β-catenin, antagonizes adipogenesis, and inhibits PPARγ transactivation, thereby maintaining MSCs in a multipotent state primed for osteoblast differentiation [151, 156, 157]. Collectively, exercise-induced mechanical loading suppresses PPARγ through multiple pathways, providing a molecular basis for its role in reducing fat accumulation and promoting bone formation.

#### Lipid metabolism regulation by shear stress and PPARγ in vascular ECs

ECs are continuously exposed to blood-flow-generated shear stress, a mechanical cue essential for vascular homeostasis. Under hypertensive or disturbed-flow conditions, mechanical stretching downregulates PPARγ in ECs. PPARγ downregulation alters cell phenotype and may indirectly affect lipid-metabolism gene regulation, thereby modifying the lipid transport and deposition environment within the vessel wall [158–160]. Thus, physiological laminar shear stress likely sustains normal PPARγ expression to exert anti-inflammatory and anti-fibrotic effects, whereas pathological disturbed flow promotes vascular dysfunction partly by suppressing PPARγ.

### Aberrant regulation under pathological conditions

In various metabolic diseases, the mechanical microenvironment of tissues undergoes significant alterations (e.g. as increased stiffness and abnormal mechanical stress). These aberrant mechanical signals and PPARγ dysfunction intertwine to jointly drive disease progression.

#### Impact of altered mechanical microenvironment on PPARγ and lipid metabolism in obesity

Obesity is associated with adipose tissue fibrosis and increased matrix stiffness [161, 162]. Obesity manifests as adipose tissue expansion and changes in its physical properties, notably, a substantial increase in matrix stiffness. A stiffened microenvironment produces an aberrant mechanical signal. In 3D scaffolds constructing microenvironments of varying stiffness, a lower mechanical stiffness promotes adipose tissue formation [117, 163–166]. This suggests that in the early stages of obesity, a softer substrate may permit sufficient PPARγ activation to promote adipocyte differentiation for accommodating excess energy through mechanisms possibly involving the YAP/TAZ pathway. However, as adipose tissue continuously expands and undergoes fibrosis, increasing matrix stiffness may suppress PPARγ activity by activating mechanotransduction pathways, such as YAP/TAZ. This leads to adipocyte dysfunction and pro-inflammatory cytokine release, thereby initiating a vicious cycle.

#### Association between disrupted mechanical signals and PPARγ dysfunction in fatty liver disease

MASLD is characterized by excessive hepatic lipid accumulation and increased liver stiffness [167]. This pathological mechanical signal can activate mechanosensing pathways, including YAP/TAZ [168], which in turn may suppress PPARγ activity, impairing its regulatory capacity over fatty acid synthesis and exacerbating lipid deposition. The traditional Chinese medicine compound, Xiaozhi Fang (XZF), improves MASLD by enhancing p-AMPK and PPARα expression, and inhibits PPARγ expression [169], suggesting that changes in liver stiffness might indirectly affect PPARγ function through AMPK/PPAR signaling axis disruption. From a mechanical signaling perspective, it can be speculated that alterations in liver stiffness might indirectly influence PPARγ function via disruption of the AMPK/PPAR axis. The mechanism by which liver stiffness regulates PPARγ activity through mechanotransduction pathways (e.g., integrins and FAK) and its role in MASLD pathogenesis require further investigations.

#### Lipid deposition regulation by the mechanical signal-PPARγ axis in cardiovascular diseases

During atherosclerotic plaque formation, mechanical stress on the vascular wall significantly increases. Atherosclerosis is characterized by lipid accumulation and chronic inflammation in the arterial wall [170, 171]. This mechanical stretch reduces PPARγ protein levels via NEDD4-mediated ubiquitination and degradation [16]. This promotes EndMT and weakens PPARγ gene regulation related to lipid metabolism, exacerbating lipid deposition and inflammation. PPARγ inactivation directly promotes EndMT, which is an important source of vascular fibrosis. This provides direct molecular evidence that PPARγ dysfunction leads to tissue fibrosis and further increases mechanical stiffness. Targeted nanoparticle drug delivery (MMR-Lobe-Cy) of the PPARγ agonist rosiglitazone to macrophages can activate PPARγ and suppress the Toll-Like Receptor 4 (TLR4)/NF-κB pathway, alleviating plaque inflammation [172–174]. This indicates that restoring PPARγ function can stabilize plaques even in a high mechanical stress environment, suggesting the mechanical signal-PPARγ axis could be a novel therapeutic target for cardiovascular diseases.

#### Mechanical signal-PPARγ regulatory mechanism in diabetes-related lipid metabolism disorders

Diabetes and its complications are often accompanied by severe lipid metabolism disorders, in which mechanical signals play an important role. In diabetic sarcopenia, aberrant STING pathway activation exacerbates skeletal muscle atrophy [175, 176], whereas a decline in PPARγ protein level is a critical driver of muscle atrophy, and its stability is regulated by USP2-mediated deubiquitination [177]. Although direct evidence linking STING to the ubiquitin-dependent degradation of PPARγ is currently lacking, it is plausible that crosstalk between these two pathways, in the context of lipid metabolic disturbances, synergistically contributes to the pathogenesis of sarcopenia. Electroacupuncture (EA) remodels glucose and lipid metabolic homeostasis by activating the PPARγ signaling pathway and its downstream cholesterol efflux targets, including ABCA1 and ABCG1, in high-fat diet-induced hyperlipidemic rats [178]. Meanwhile, the shear stress generated by acupuncture can be sensed by mechanosensitive channels such as Piezo1 and transient receptor potential cation channel subfamily V member 4 (TRPV4) on macrophages; activation of these channels induces intracellular Ca^2^⁺ fluctuations and promotes YAP/TAZ nuclear translocation, thereby converting physical stimuli into biochemical responses [179]. Based on these findings, we hypothesize that EA may activate mechanosensitive channels to drive YAP/TAZ nuclear translocation, thereby acting as transcriptional coactivators to regulate PPARγ activity and function of at the transcriptional level. However, the proposed mechanical mechanism remains theoretical at present, and further experimental studies are needed to validate it.

Across various metabolic diseases, the relationship between aberrant mechanical signals and PPARγ dysfunction is fundamentally cyclical. The initial pathological insult, whether nutrient excess in obesity, hemodynamic disturbance in atherosclerosis, or metabolic stress in diabetes, leads to both tissue mechanical alteration (e.g., fibrosis, stiffening) and PPARγ dysregulation [16, 17, 161, 175]. Critically, these two events are causally linked: altered mechanics suppress PPARγ, and PPARγ dysfunction promotes further mechanical remodeling (e.g., by facilitating fibrosis). This vicious cycle establishes a self-perpetuating pathological state in which PPARγ, acting as a mechano–metabolic transducer, becomes trapped in a dysfunctional loop, driving progressive tissue failure and metabolic deterioration.

For obesity, MASLD, and atherosclerosis, considerable evidence from in vitro and animal studies supports a causal role of mechanical microenvironment alterations in PPARγ dysfunction [16, 122, 169]. In contrast, for diabetes and its complications (e.g., sarcopenia and cardiomyopathy), the association between mechanical signals and PPARγ remains largely correlative or relies on indirect evidence, such as pharmacological inhibitor studies [175–177]. Furthermore, clinical evidence directly testing whether restoration of normal tissue mechanics improves PPARγ function is currently lacking. Therefore, until further evidence from mechanistic studies and clinical trials is obtained, the therapeutic potential of mechanical interventions remains to be determined, and their definitive efficacy requires confirmation through additional research.

Collectively, PPARγ is a central hub through which mechanical signals regulate lipid metabolism. Under physiological conditions, appropriate mechanical loading helps maintain energy balance and tissue homeostasis by suppressing PPARγ. In contrast, under pathological conditions, an aberrant mechanical microenvironment and PPARγ dysfunction interact, jointly driving the progression of various metabolic diseases, such as obesity, fatty liver, atherosclerosis, and diabetes. A deeper understanding of the bidirectional regulatory mechanisms within this “mechano-PPARγ” axis will provide an important theoretical foundation for developing novel metabolic disease therapies based on physical interventions or targeting mechanotransduction pathways.

## Therapeutic implications of targeting the mechano‑PPARγ axis

PPARγ is a major therapeutic target for metabolic diseases, with TZDs as the prototype agonists [56, 180]. Recently, PPARγ-targeted drug development has progressed from simple agonism or antagonism toward more precise, tissue-selective, and functionally specific modulation. This evolution encompasses natural compounds, structurally optimized synthetic ligands, nanotechnology-based delivery systems, and artificial intelligence-guided designs (Table 1), all aimed at precisely tuning PPARγ activity to counteract pathologies driven by the combined mechanical microenvironment dysregulation and PPARγ function. These emerging strategies focus on maximizing beneficial effects of PPARγ, such as improving insulin sensitivity, regulating lipid metabolism, and exerting anti-inflammatory action, and minimizing adverse effects, such as weight gain, fluid retention, and abnormal bone metabolism [181]. Consequently, developing agents that restore or enhance PPARγ function, particularly in aberrant mechanical settings, is a promising therapeutic avenue for obesity, atherosclerosis, fatty liver disease, and diabetes. However, the efficacy and safety of these agents are profoundly influenced by the mechanical microenvironment of the target tissue. In this section, we reorganize the therapeutic strategies according to major metabolic diseases, explicitly linking each disease to its characteristic mechanical signals, mechanosensors, downstream pathways, PPARγ regulation, lipid consequences, and clinical evidence.

**Table 1:** Applications and clinical progress of PPARγ agonists in various diseases

Drug/Intervention Name	Type	Target Disease	Effect on PPARγ	Main Outcomes	Clinical Progress
Curcumin	Natural agonist	Inflammatory diseases (IBD, arthritis, atherosclerosis); cardiac fibrosis; MASLD; T2D	Activates PPARγ, inhibits NF-κB; upregulates PPARγ to inhibit TGF-β/Smad [182]	Anti-inflammatory (↓TNF-α, IL-6, IL-1β); anti-fibrotic; antioxidant; anti-atherosclerotic	Clinical trials demonstrate improvement in inflammatory markers and symptoms, yet direct evidence of PPARγ expression/activity in humans remains absent [183]
Quercetin	Natural agonist	Atherosclerosis, cardiovascular disease	Upregulates PPARγ; activates PPARγ-LXRα-ABCA1 reverse cholesterol transport pathway	Promotes macrophage cholesterol efflux; reduces foam cell formation; anti-atherosclerotic	Preclinical studies [184, 185]
Resveratrol	Natural agonist	Obesity, MASLD, insulin resistance, metabolic syndrome	Activates PPARγ; improves adipose tissue function; regulates adipokine secretion	Reduces hepatic steatosis; improves insulin sensitivity; anti-inflammatory and antioxidant	Preclinical animal study (rat model) [186]
Conjugated linoleic acid	Natural agonist	Obesity, diabetic cardiomyopathy	Activates PPARγ to promote adipocyte differentiation; PPARγ-dependent and -independent mechanisms [187, 188]	Reduces body fat; promotes browning (UCP1↑); protects cardiomyocyte contractile function	Reduced total and abdominal fat in healthy postmenopausal women (NCT00474552, completed, Denmark). While not directly linked to PPARγ, the fat reduction suggests a potential relationship with PPARγ [189]
Honokiol	Natural agonist (dual PPARα/γ)	MASLD, obesity	Dual PPARα/γ agonist; activates PPARγ to promote UCP1 expression and browning	Promotes fatty acid oxidation; increases energy expenditure; anti-obesity; reduces hepatic steatosis	Preclinical studies (mouse models and cell experiments) [190]
Magnolol	Natural agonist (dual PPARα/γ)	MASLD, obesity	Dual PPARα/γ agonist	Promotes fatty acid oxidation and browning; anti-obesity; liver protection	Preclinical studies [190]
Micheliolide	Natural agonist	MASLD/NASH, hepatic steatosis	Upregulates PPARγ; inhibits NF-κB and activates AMPK/mTOR autophagy pathway via PPARγ	Reduces hepatic inflammation and steatosis; enhances autophagy (↑LC3B-II/I); reduces liver lipids and transaminases	mouse model and hepatocyte cell line experiments [191]
Amorfrutins	Natural selective PPARγ modulator	T2D, insulin resistance, metabolic syndrome	Directly binds and selectively activates PPARγ (different cofactor recruitment from TZDs)	Improves insulin sensitivity; lowers blood glucose; increases adiponectin secretion; no weight gain	Preclinical stage (in vitro and mouse studies) [192]
Berberine	Natural agonist	T2D, MASLD, obesity, insulin resistance	Activates PPARγ; improves insulin signaling and lipid metabolism [193]	Lowers blood glucose and lipids; improves insulin resistance; modulates gut microbiota	Reduce the glucose & lipids in T2D + dyslipidemia (NCT00462046, completed, China); no direct human PPARγ evidence [194]
Falcarindiol/Falcarinol	Natural agonist	T2D, insulin resistance	Partially activates PPARγ to promote glucose uptake	Increases glucose uptake in adipocytes and myotubes	In vitro basic research stage [195]
Vitamin E	Natural antioxidant	MASLD/NASH	Indirectly regulates PPARγ-related oxidative stress pathways	Antioxidant effect; improves NASH histology	Improves histology in non‑diabetic NASH (NCT00063622, completed, USA) but fails in T2D + NASH (NCT01002547, terminated, USA); no direct human PPARγ evidence [196, 197]
Pioglitazone	Synthetic agonist (TZD class)	T2D, insulin resistance, MASLD/NASH, cardiovascular disease	Full PPARγ agonist; promotes adipocyte differentiation; improves insulin sensitivity	Lowers blood glucose; improves lipid profile (↓TG, ↑HDL-C); reduces visceral fat; reduces macrovascular events	Improves T2D glycemia & insulin sensitivity via PPARγ (NCT00174993, completed, multinational) [198]
Rosiglitazone	Synthetic agonist (TZD class)	T2D, insulin resistance, MASLD	Full PPARγ agonist; insulin sensitizer	Improves insulin resistance; lowers FFA and TG; weight gain and edema (side effects)	Improves insulin sensitivity & glycemic control in T2D (NCT00174993, completed, multinational) [199]
Lobeglitazone	Synthetic agonist (TZD class)	T2D	PPARγ agonist; insulin sensitizer	Improves glycemic control	Improves glycemia & lipid profiles in T2D (NCT01001611, completed, South Korea) [200]
Saroglitazar	Synthetic agonist (dual PPARα/γ)	MASLD/NASH, diabetic dyslipidemia	Dual PPARα/γ agonist	Improves hepatic steatosis and fibrosis; regulates lipid metabolism	Improved ALT, liver fat, insulin resistance, and dyslipidemia in MASLD/NASH patients, regardless of comorbidities and statin use (NCT03061721, Phase II; NCT03863574, Phase II, all completed, USA). Direct via PPARγ (dual PPARα/γ agonist) [201]
Elafibranor	Synthetic agonist (dual PPARα/δ)	MASLD/NASH	PPARα/δ agonist (not directly targeting PPARγ, but pathway-related)	Improves hepatic steatosis, inflammation, and fibrosis	Improves biochemical response and normalization of alkaline phosphatase in patients with primary biliary cholangitis; no direct human PPARγ evidence (NCT04526665, Phase III, completed, multinational, including US) [202]
Resmetirom (MGL-3196)	Synthetic agonist (THRβ-selective)	MASLD/NASH	THRβ agonist (cross-regulates hepatic lipid metabolism with PPARγ pathway) [203]	Significantly reduces liver fat content; improves NASH histology	Improves NASH resolution & fibrosis; no direct human PPARγ evidence (NCT03900429, Phase III, completed, multinational; FDA accelerated approval 2024–03–14) [204]
GLP-1 analogs (Semaglutide, etc.)	Synthetic agonist (GLP-1 receptor)	T2D, obesity, MASLD	Indirectly cross-regulates with PPARγ pathway	Significantly reduces body weight and HbA1c; improves liver fat content	Improve NASH resolution, reduce weight and cardiometabolic risk factors in NASH/obesity; no direct human PPARγ evidence (NCT01237119, Phase II; NCT03548933, Phase III; NCT02970942, Phase II; all completed, multinational, UK) [205–207]
TZDs	Synthetic agonist (full PPARγ agonist)	T2D, MASLD/NASH	Full PPARγ agonist; promotes adipocyte differentiation; improves insulin sensitivity [203, 208, 209]	Reduces hepatic steatosis; improves insulin resistance and glycemic control; weight gain and edema (side effects)	Improve liver histology, ALT, fat, insulin resistance, dyslipidemia in NASH/MASLD (NCT00063622, Phase II; NCT03061721, Phase II, all completed, USA), effects direct via PPARγ. MSDC-0602K (PPARγ-sparing): improves steatosis (NCT02784444, Phase II, USA) via MPC inhibition, not direct PPARγ [210]
Metformin	Synthetic agonist (AMPK activator)	T2D, obesity, MASLD	Indirectly regulates PPARγ-related metabolic pathways (via AMPK activation)	Lowers blood glucose; improves insulin resistance; mild weight loss	Reduced HbA1c (−1.48%) and body weight (−2.0 kg) in drug-naive patients with T2D, yet direct evidence of PPARγ expression/activity in humans remains absent (NCT00676338, Phase III, completed, multinational) [211]

*PPARα* peroxisome proliferator-activated receptor alpha, *PPARγ* peroxisome proliferator-activated receptor gamma, *Smad* (Sma- and Mad-related protein), *TGF-β* transforming growth factor-beta; *IBD* Inflammatory Bowel Disease, *NF-κB* Nuclear Factor kappa B, *TNF-α* Tumor necrosis factor-alpha, *IL-6* Interleukin-6, *IL-1β* Interleukin-1 beta, *MASLD* metabolic dysfunction-associated steatotic liver disease, *LXRα* liver X receptor alpha, *ABCA1* ATP-binding cassette transporter A1, *UCP1* uncoupling protein 1, *AMPK* AMP-activated protein kinase, *TZD* thiazolidinedione, *T2D* type 2 Diabetes, *FFA* Free Fatty Acids, *TG* triglyceride, *HDL-C* High-Density Lipoprotein Cholesterol, *ALT* Alanine Aminotransferase, *THRβ* Thyroid Hormone Receptor beta, *GLP-1* Glucagon-like Peptide-1, *HbA1c* Hemoglobin A1c, *MPC* Mitochondrial Pyruvate Carrier, *NCT* National Clinical Trial, *NASH* Non-Alcoholic Steatohepatitis. ↑, increase; ↓, decrease

### Obesity

In obesity, progressive adipose tissue expansion leads to increased matrix stiffness and tissue fibrosis, which activate the integrin–FAK–YAP/TAZ mechanotransduction axis [212, 213]. Adipose tissue fibrosis is now recognized as a hallmark of the obese state and directly contributes to the pathogenesis of obesity-related metabolic complications [214]. Elevated pressure within orbital adipose tissue also activates Piezo1 channels [113]. Soft, compliant adipose tissue permits PPARγ expression and adipogenesis, whereas stiffening suppresses PPARγ via YAP/TAZ, impairing adipocyte function and promoting inflammation and lipid spillover [45, 122, 215]. Consequently, a vicious cycle is established in which fibrosis leads to increased stiffness, which in turn suppresses PPARγ and ultimately drives metabolic dysfunction [45, 161, 216]. Several PPARγ‑targeted agents have been tested in obesity. The TZDs pioglitazone and rosiglitazone improve insulin sensitivity by activating PPARγ [217], but their long‑term efficacy may be reduced in fibrotic, stiff adipose tissue. Pioglitazone is FDA‑approved for T2D and shows benefit in obesity‑related metabolic dysfunction, though weight gain remains a concern [196]. Due to cardiovascular safety concerns, rosiglitazone was withdrawn in Europe in 2010 and has restricted use in some regions [208, 209]. Lobeglitazone, another TZD, is approved in South Korea for T2D [209]. Selective PPARγ modulators such as INT131 aim to preserve insulin sensitization while avoiding weight gain and edema [218]. Natural agonists have also been explored. Conjugated linoleic acid (CLA) activates PPARγ to promote adipocyte differentiation and browning (UCP1 upregulation) and reduces body fat in animal studies [187, 188]. The same pattern has also been found in human clinical studies. Resveratrol activates PPARγ, improves adipose tissue function and reduces hepatic steatosis, though its low bioavailability limits clinical application [186]. Magnolol and honokiol, dual PPARα/γ agonists, promote fatty acid oxidation and energy expenditure, counteracting obesity in preclinical models [190]. Berberine activates PPARγ via the AMPK/SIRT1 pathway, improves insulin resistance and promotes white adipose tissue browning, and is available as an over‑the‑counter supplement in some countries [219]. Physical interventions such as low‑energy shockwave therapy suppress PPARγ‑driven adipogenesis via the cAMP/PKA/β‑catenin axis, reducing local fat deposition [127]. Low‑magnitude whole‑body vibration (0.3 g, 90 Hz) downregulates PPARγ in bone marrow MSCs and prevents diet‑induced obesity [45, 153]. Mechano‑responsive nanoparticles that release PPARγ agonists only under pathological stiffness are being explored to enhance targeting and reduce systemic side effects. From the existing data, one can infer that PPARγ activation is effective in early, non‑fibrotic adipose tissue. However, it cannot be inferred that PPARγ agonists are efficacious in advanced, stiff, fibrotic obesity, nor are the long‑term safety and optimal parameters of mechanical interventions known.

### Atherosclerosis and cardiovascular diseases

Atherosclerosis is a chronic inflammatory disease of the arterial wall, and its localization to specific regions of the vasculature is determined by local hemodynamic forces, in particular, the shear stress generated by blood flow [220]. In atherosclerosis and cardiovascular diseases, ECs in atheroprone regions are exposed to low or impinging shear stress, which activates PKCα‑ERK signaling and suppresses PPARγ transcription [17]; in parallel, sustained mechanical stretch (as in portal hypertension) triggers ROS/NF‑κB‑dependent NEDD4 upregulation and subsequent PPARγ ubiquitination [16]. Impairment of PPARγ function leads to endothelial‑to‑mesenchymal transition [16], upregulation of NF‑κB and MMP2, and heightened inflammatory responses [17]. PPARγ downregulation also impairs reverse cholesterol transport by reducing ABCA1/ABCG1 expression [55, 221]. TZDs (pioglitazone, rosiglitazone) have anti‑atherosclerotic effects by improving endothelial function, reducing inflammation and promoting cholesterol efflux [55, 221, 222]; pioglitazone reduces major adverse cardiovascular events in high‑risk patients (PROactive study HR 0.84), but its use is limited by fluid retention and heart failure risk [198, 223]. Natural agonists also show promise. Curcumin activates PPARγ and suppresses NF‑κB, reducing pro‑inflammatory cytokines and slowing atherosclerotic plaque progression in animal models [183]. Resveratrol has anti‑inflammatory and antioxidant properties in animal models, but human data are limited [186]. Laminar shear stress (exercise‑induced) activates PPARγ via endogenous ligand production [158], upregulates its target gene *stearoyl-CoA desaturase 1 (SCD1)* [159], and induces the PPARγ‑cytochrome P450 family 27 (CYP27)‑LXR‑ABCA1 pathway to promote cholesterol efflux [160], thereby maintaining vascular homeostasis. Targeted delivery of the PPARγ agonist lobeglitazone via macrophage‑targeted nanoparticles (MMR‑Lobe‑Cy) has been shown to stabilize plaques and suppress TLR4/NF‑κB in preclinical models [172]. One can infer that PPARγ activation is protective in regions with laminar shear stress and that TZDs are effective in reducing systemic atherosclerosis. What cannot be inferred is whether PPARγ agonists are effective in vessels exposed to impinging or OSS (e.g., coronary bifurcations, carotid sinuses), or whether NEDD4 inhibitors could synergize with TZDs in these regions.

### Metabolic Dysfunction‑Associated Fatty Liver Disease (MASLD)

MASLD is characterized by hepatic steatosis, which may progress to nonalcoholic steatohepatitis, cirrhosis, and hepatocellular carcinoma [224]. In MASLD, progressive hepatic fibrosis and increased liver stiffness activate integrin–FAK–YAP/TAZ and Piezo1–ERK1/2 pathways [28, 225]. In early steatosis, PPARγ is upregulated and promotes lipogenesis, contributing to lipid accumulation; in advanced fibrosis, increased stiffness suppresses PPARγ transcription via YAP/TAZ, worsening insulin resistance and inflammation [226, 227]. PPARγ also transrepresses NF‑κB, limiting pro‑inflammatory cytokine release. Several PPARγ‑targeted and related agents have been evaluated in MASLD. Pioglitazone reduces hepatic triglyceride content by redistributing fat to adipose tissue and improving adiponectin levels. Pioglitazone is recommended for patients with biopsy‑proven Non-Alcoholic Steatohepatitis (NASH) but not for those with advanced cirrhosis [203]. Vitamin E, an antioxidant that indirectly modulates PPARγ‑related oxidative stress, has been used clinically for non‑diabetic NASH patients [203, 228]. Dual PPARα/γ agonists such as saroglitazar have shown promising results in clinical trials for NASH, including improvements in hepatic steatosis, inflammation, and histological markers of fibrosis [210]. Elafibranor (PPARα/δ agonist) and resmetirom (THRβ agonist, the first FDA‑approved NASH drug) also cross‑regulate lipid metabolism pathways that intersect with PPARγ [229–233]. Natural compounds including magnolol and honokiol activate both PPARα and PPARγ, improving liver‑adipose communication and attenuating steatosis [190]; micheliolide upregulates PPARγ and suppresses NF‑κB while activating AMPK/mTOR autophagy pathways, reducing hepatic lipid accumulation and inflammation in db/db mice [191]. Glucagon-like peptide-1 (GLP-1) analogues (semaglutide) and dual glucose-dependent insulinotropic polypeptide (GIP)/GLP-1 agonist tirzepatide have significantly reduces body weight and liver fat content in clinical trials, and are now approved for T2D and obesity, with ongoing NASH studies [203, 207, 234]. Metformin, an AMPK activator, is a first‑line oral glucose‑lowering agent but has shown limited efficacy in improving NASH histology [211, 235]. Exercise and mechanical loading may improve liver stiffness and restore PPARγ sensitivity, but direct evidence remains limited. Thus, PPARγ agonists are beneficial in non‑cirrhotic MASLD, but their efficacy and safety in cirrhotic patients with high stiffness remain unknown, and YAP/TAZ inhibitors have not been tested in MASLD.

### T2D and diabetic complications

In T2D and its complications, chronic hyperglycemia, oxidative stress, and altered tissue mechanics (e.g., increased cardiac stiffness in diabetic cardiomyopathy and reduced muscle tone in sarcopenia) lead to aberrant STING and AMPK/EZH2 pathway activation [146, 175, 190]. In diabetic sarcopenia, STING activation promotes PPARγ ubiquitination and degradation, impairing fatty acid oxidation and exacerbating muscle atrophy [175–177]. In diabetic myocardial fibrosis, high glucose suppresses PPARγ via AMPK/EZH2, promoting fibroblast‑to‑myofibroblast transition [146]. Numerous PPARγ‑targeted and metabolism‑modulating agents are used in T2D. TZDs (pioglitazone, rosiglitazone, lobeglitazone) improve insulin sensitivity by upregulating GLUT-4 and increasing adiponectin; pioglitazone remains widely used, whereas rosiglitazone has restricted use due to cardiovascular safety concerns [236–238]. Selective PPARγ modulators such as amorfrutins directly bind PPARγ with a distinct co‑factor recruitment pattern, improving insulin sensitivity without weight gain in preclinical models [239]. Falcarindiol and falcarinol from carrots partially activate PPARγ to enhance glucose uptake in adipocytes and myotubes [195]. Berberine, an AMPK activator that also promotes PPARγ deacetylation via SIRT1, lowers blood glucose and lipids in clinical studies, and is available as an OTC supplement in some countries [194, 219, 240]. Metformin activates AMPK and indirectly modulates PPARγ pathways, but despite preclinical benefits against HFD-induced liver fibrosis, its clinical efficacy for NASH histology remains uncertain [241–243]. GLP‑1 analogues (semaglutide) and tirzepatide (GIP/GLP‑1 dual agonist) produce substantial weight loss and glycemic control and are approved for T2D and obesity, with ongoing trials in NASH. Electroacupuncture activates PPARγ and improves glucose‑lipid homeostasis, possibly by modulating tissue mechanical properties, such as muscle tone and blood flow [244–248]. Exercise and mechanical loading restore normal tissue mechanics and may sensitize tissues to PPARγ activation. PPARγ activation improves systemic insulin resistance and lipid profiles in T2D. However, whether mechanical interventions (electroacupuncture, exercise) act directly via PPARγ or through other pathways cannot be established, nor has the efficacy of PPARγ agonists been specifically tested in diabetic sarcopenia or cardiomyopathy.

### Translational evidence and challenges for mechanical interventions

Beyond pharmacological agents, physical interventions that directly apply mechanical cues to target tissues have emerged as potential non‑invasive strategies for managing obesity and metabolic disorders. Preclinical studies have established that low-energy shockwave therapy suppresses adipogenesis via the cAMP/β-catenin/PPARγ pathway in 3T3-L1 cells and human primary preadipocytes [127]. In humans, small randomized trials have shown that shockwave therapy combined with topical retinol significantly reduces subcutaneous fat thickness, and that variable-frequency whole-body vibration combined with dietary intervention decreases waist circumference (approximately −9.6 cm) and visceral adipose tissue (−47.8 cm^2^) in overweight and obese adults [249, 250]. Nevertheless, several major challenges remain: lack of standardized protocols (optimal frequency, intensity, duration, number of sessions), safety concerns (local pain, bruising, nerve irritation; contraindications for patients with bleeding disorders, malignancies or pregnancy), patient variability and adherence issues, high device costs and limited insurance coverage, and the absence of long‑term safety and efficacy data. Future research must focus on dose‑response studies to establish standardized protocols, large‑scale long‑term RCTs in diverse populations, identification of responder biomarkers, and development of portable low‑cost wearable devices.

Notably, the mechanical microenvironment profoundly influences the efficacy and safety of PPARγ agonists and modulators. The response to these agents varies substantially depending on the local mechanical context. As summarized in Table 2, distinct mechanical signals, such as mechanical stretch (portal hypertension), matrix stiffness, mechanical overload, mechanical compression, cyclic stretch, low‑magnitude mechanical signals, and mechanical strain, activate specific mechanosensors and downstream pathways, leading to differential regulation of PPARγ expression, activity, or post‑translational modifications. For instance, under mechanical stretch mimicking portal hypertension, NEDD4‑mediated ubiquitination and degradation of PPARγ promote EndMT and vascular dysfunction, suggesting that PPARγ agonists, such as rosiglitazone, could be repurposed to mitigate portal hypertension‑induced endothelial injury [16]. Increased matrix stiffness promotes Pyruvate kinase M2 (PKM2) dimer formation and their nuclear translocation with YAP, which suppresses PPARγ expression and drives myofibroblast activation, a process that can be reversed by the adipokine Omentin-1 through restoration of PPARγ activity [77]. Mechanical overload in tendons activates mTOR signaling and promotes aberrant non-tenogenic differentiation of tendon stem/progenitor cells, which can be prevented by the mTOR inhibitor rapamycin [145]. In a separate context, mechanical loading (axial loading) downregulates PPARγ and C/EBPα to reduce bone marrow adiposity, while also suppressing receptor activator of nuclear factor-κB ligand (RANKL)-mediated osteoclastogenesis, thereby attenuating bone resorption in obesity-associated breast cancer bone metastasis [251]. Mechanical compression suppresses PPARγ2 expression via a Cyclooxygenase-2 (COX‑2)‑dependent pathway, inhibiting adipogenic differentiation of adipose‑derived stem cells, providing a cellular basis for physical interventions such as massage or pressure garments in obesity management [252, 253]. Each of these mechanical contexts leads to distinct metabolic or pathological outcomes and accordingly suggests different therapeutic opportunities, such as using PPARγ agonists, YAP inhibitors, mTOR inhibitors, mechanical loading, or compression therapy. Therefore, when evaluating PPARγ‑targeted therapies, the characteristic mechanical signals present in each disease state should be carefully considered. Table 2 provides a summary of key mechanotransduction pathways and their corresponding therapeutic implications, serving as a practical guide for context‑dependent drug development and physical intervention strategies.

**Table 2:** Mechanistic insights into mechanical signal-mediated PPARγ regulation and therapeutic implications

Mechanical cue	Cell/Tissue Type	Upstream Mechanosensor	Signaling Pathway	Effect on PPARγ	Metabolic/Pathological Outcome	Therapeutic Implication
Pressure overload (hypertension)	Cardiac fibroblasts, left ventricle of SHRs	AT1R	PPARγ activation inhibits TGF-β1/Smad2/3	expression ↑ PPREs-binding activity↑	Attenuation of cardiac fibrosis; decreased collagen deposition; reduced heart weight/body weight ratio	Curcumin provides a natural therapeutic strategy for hypertensive cardiac fibrosis by activating PPARγ and inhibiting the TGF-β1/Smad2/3 pathway [182]
Mechanical stretch (portal hypertension)	HUVECs, portal vein endothelium of cirrhotic rats	Not specified	PI3K/AKT/CREB inactivation- PPARγ↓; NEDD4-mediated PPARγ degradation; promote Smad3 phosphorylation	transcriptional repression↓ ubiquitination/degradation↑	EndMT; vascular fibrosis	PPARγ agonists (e.g., rosiglitazone) may serve as a drug repurposing candidate for portal hypertension by inhibiting EndMT and alleviating portal vein endothelial injury [16]
Matrix stiffness (ECM stiffness)	Lung fibroblasts (myofibroblasts)	Intergrin	PKM2 dimerization causes YAP nuclear translocation	Upregulation and activation (phosphorylation at Ser112)	Myofibroblast-to-lipofibroblast conversion; reduced collagen synthesis, increased collagen degradation; fibrosis resolution	The adipokine omentin-1 may provide a novel therapeutic strategy for IPF by restoring PPARγ activity and promoting myofibroblast-to-lipofibroblast conversion、 [77]
Mechanical overloading (tendon overuse)	TSPCs	Not specified	mTOR activation causes non-tenocyte differentiation	Upregulation	Tendinopathy (round cells, proteoglycan accumulation, expression of SOX-9/collagen II)	The mTOR inhibitor rapamycin may prevent overuse-induced tendinopathy by blocking mechanical overloading-induced non-tenocyte differentiation [145]
Mechanical overloading (diabetic tendon)	Rat primary tenocytes	Not specified	ERK activation and Akt inactivation downregulate PPARγ and adipogenic genes	Downregulation	Prevents adipogenic transdifferentiation; maintains fibroblastic phenotype and migration	Cyclic stretching exercise may serve as an adjunct therapy for diabetic tendinopathy by inhibiting PPARγ-mediated adipogenic transdifferentiation of tenocytes [254]
Mechanical loading (axial loading)	Bone marrow‑derived cells (osteoclasts, osteoblasts, adipocytes)	Not specified	RANKL/CTSK (osteoclast) ↓ PPARγ/C/EBPα (adipocyte) ↓ OPG (osteoblast) ↑	Downregulation	Reduced osteolysis, improved bone microarchitecture, inhibited tumor growth/metastasis	Mechanical loading as non‑invasive palliative therapy for breast cancer bone metastasis, especially in obesity by downregulating the PPARγ-RANKL axis and inhibiting tumor-induced bone destruction [251]
Mechanical compression (cyclic compressive force)	ASCs	Not specified	PPAR‑γ expression (COX‑2‑dependent pathway suggested) ↓	Downregulation	Reduced adipogenesis, fewer lipid droplets	Mechanical compression (massage, pressure garments) may help prevent obesity by inhibiting adipogenic differentiation of ASCs [252]
Low-magnitude mechanical signals (whole body vibration)	Bone marrow-derived MSCs	Not specified	Upregulation of Runx2 (osteogenic marker) and downregulation of PPARγ (adipogenic marker)	Downregulation	Increased trabecular bone volume, decreased visceral fat	Non‑pharmacologic strategy to prevent osteoporosis and obesity simultaneously [153]
Mechanical strain (cyclic loading)	Mouse MSCs	Not specified	β‑catenin activation reduces PPARγ2 expression and suppresses its transcriptional activity	Downregulation and functional inhibition	Preserved MSC multipotentiality, reduced adipogenesis, enhanced osteogenic response to BMP‑2	Mechanical loading (exercise) to counteract marrow adiposity and preserve bone health [6]
Impinging flow	HUVECs	Not specified (Ca^2^⁺ influx involved)	Ca^2^⁺ → PKCα activation → ERK5/ERK1/2 phosphorylation → PPARγ ↓ → NF‑κB/MMP2 ↑	Downregulation	Endothelial inflammation (NF-κB ↑), ECM degradation (MMP2 ↑), apoptosis; contributes to intracranial aneurysm formation	Targeting PKCα/ERK/PPARγ pathway may alleviate flow-induced endothelial injury; PPARγ agonists (e.g., PGZ) show protective effects [17]
Low-energy shockwave	3T3-L1 preadipocytes, human primary subcutaneous preadipocytes	Not specified	Shockwave causes extracellular ATP release, activates Wnt10b/β-catenin, suppresses PPARγ, and C/EBPα	Downregulation	Inhibition of adipocyte differentiation; reduce intracellular lipid droplet accumulation; suppress expression of adipogenic markers	Low-energy shockwave treatment may serve as a non-invasive strategy to inhibit adipogenesis and combat obesity by downregulating PPARγ [127]

*PPARγ* peroxisome proliferator-activated receptor gamma, *Smad* (Sma- and Mad-related protein), *TGF-β* transforming growth factor-beta, *ECM* Extracellular matrix, *COX-2* Cyclooxygenase-2, *NF-κB* Nuclear Factor kappa B, *PKM2* Pyruvate kinase M2, *RANKL* receptor activator of nuclear factor-κB ligand, *YAP* Yes-associated protein, *PI3K* Phosphoinositide 3-kinase, *SOX9* SRY-box transcription factor 9, *Runx2* Runt-related transcription factor 2, *CTSK* Cathepsin K, *PKCα* protein kinase C alpha, *MMP2* matrix metallopeptidase 2, *OPG* Osteoprotegerin, *BMP‑2* Bone morphogenetic protein 2, *SHRs* spontaneously hypertensive rats, *AT1R* Angiotensin II receptor, *PPREs* Peroxisome proliferator response element, *HUVECs* Human umbilical vein endothelial cells, *CREB* cyclic-AMP response element-binding protein, *mTOR* mammalian Target of Rapamycin, *NEDD4* neural precursor cell expressed developmentally down-regulated protein 4/, *EndMT* endothelial-to-mesenchymal transition, *IPF* idiopathic pulmonary fibrosis, *TSPCs* tendon stem/progenitor cells, *ERK* extracellular signal-regulated kinase, *ASCs* adipose-derived stem cells, *MSCs* mesenchymal stem cells. **↑**, increase; **↓**, decrease

## Conclusions and perspectives

**Fig. 5:**
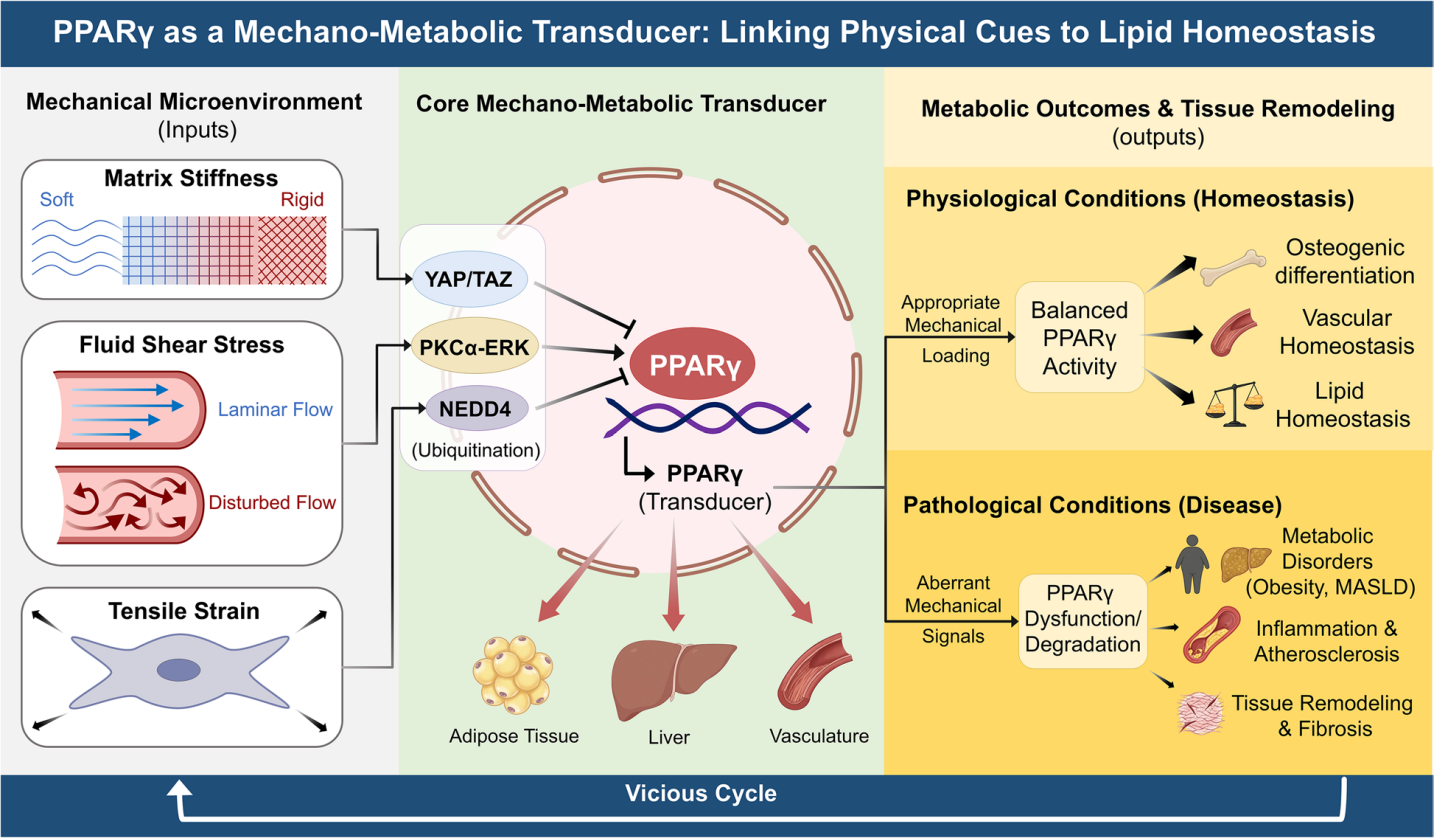
Schematic illustration of PPARγ as a central mechano‑metabolic transducer linking mechanical signals to lipid homeostasis and tissue remodeling. Multiple physical inputs from the mechanical microenvironment, including matrix stiffness (soft or stiff), fluid shear stress, and tensile strain, are integrated by cells through specific mechanosensing and signaling pathways. These signals converge on regulatory cascades involving YAP/TAZ, PKCα–ERK, and NEDD4‑mediated ubiquitination to modulate the expression and activity of PPARγ, thereby influencing the functions of major metabolic organs such as adipose tissue, liver, and vasculature. Under physiological conditions, appropriate mechanical loading maintains balanced PPARγ activity, promotes osteogenic differentiation, preserves vascular homeostasis, and sustains lipid metabolic equilibrium. In contrast, under pathological conditions, aberrant mechanical cues suppress PPARγ function or accelerate its degradation, leading to PPARγ dysfunction. This, in turn, drives the development of metabolic disorders such as obesity and non‑alcoholic fatty liver disease, and promotes pathological remodeling processes including inflammation, atherosclerosis, and tissue fibrosis. The resulting fibrosis and tissue stiffening further exacerbate abnormalities in the mechanical microenvironment, establishing a vicious cycle of “mechanical signal imbalance–metabolic dysregulation–tissue remodeling.” Abbreviations: PPARγ, peroxisome proliferator-activated receptor gamma; YAP, Yes-associated protein; TAZ, transcriptional coactivator with PDZ-binding motif; PKCα, protein kinase C alpha; ERK, extracellular signal-regulated kinase; NEDD4, neural precursor cell expressed developmentally down-regulated protein 4

Mechanical signals serve as crucial physical cues that regulate cellular functions. Through multilayered signaling networks, they precisely modulate the expression, activity, and post-translational modifications of PPARγ, thereby substantially influencing lipid metabolic homeostasis and tissue remodeling. PPARγ is no longer viewed as only a conventional nuclear receptor for metabolic regulation; it is increasingly recognized as a “mechano-metabolic transducer” that links the mechanical microenvironment to cellular metabolic reprogramming. Distinct from primary mechanosensors such as Piezo1 or integrins, PPARγ does not directly sense physical forces but rather integrates upstream mechanotransduction signals and converts them into lipid metabolic gene expression programs. Under physiological conditions, appropriate mechanical stimuli, such as exercise-associated tensile strain and laminar shear stress, guide MSCs toward osteogenic differentiation, maintain vascular endothelial homeostasis, and suppress abnormal lipid accumulation by inhibiting PPARγ activity or modulating its co-activators [151, 152, 154, 158]. In contrast, pathological mechanical environments, marked by increased tissue stiffness, disturbed blood flow, or abnormal loading, inhibit PPARγ function via pathways, including YAP/TAZ, NEDD4-mediated ubiquitination, PKCα-ERK, and cAMP/Wnt/β-catenin [14–17, 127]. This establishes a vicious cycle of mechanical imbalance, metabolic dysregulation, and tissue remodeling. The systematic dissection of the mechanical signal-PPARγ-lipid metabolism axis deepens our multidimensional understanding of metabolic disease pathogenesis and provides a novel theoretical foundation for moving beyond purely biochemical perspectives toward physical intervention strategies (Fig. 5).

However, several important questions remain. First, what is the cell‑type‑specific and spatiotemporal heterogeneity of PPARγ mechano‑responses? Although PPARγ responds to mechanical signals in adipocytes, ECs, and MSCs, systematic mapping across multiple tissues and disease stages is lacking. Integrating single‑cell multi‑omics (simultaneous scRNA‑seq and scATAC‑seq) with spatial transcriptomics on tissue sections from mechanical gradient models (e.g., stiff versus soft regions of fibrotic liver or adipose tissue) holds promise for revealing previously unrecognized PPARγ‑positive mechano‑sensitive subpopulations and their dynamic transitions. Second, do distinct mechanical signals converge on common or signal‑specific post‑translational modifications of PPARγ? Shear stress, matrix stiffness, and cyclic stretch all suppress PPARγ, but whether they utilize shared or unique post‑translational modification codes (e.g., Ser273 phosphorylation vs. Lys367 ubiquitination) remains unknown. Quantitative mass spectrometry‑based phosphoproteomics and ubiquitinomics on cells exposed to each mechanical cue, followed by in vivo validation with modification‑specific mutants, may clarify this issue. Third, which molecular nodes in the mechano‑PPARγ network are most druggable? The vicious cycle involves YAP/TAZ, NEDD4, PKCα‑ERK, and AMPK as upstream regulators. Testing existing pharmacological inhibitors (e.g., YAP‑TEAD inhibitors, NEDD4 inhibitors, AMPK activators) in preclinical models of obesity‑induced fibrosis or atherosclerosis, or developing mechano‑responsive nanoparticles that release PPARγ agonists only under pathological stiffness conditions, may improve targeting and reduce systemic side effects. Fourth, can breaking the mechanical feedback loop reverse established metabolic disease? Current studies correlate tissue stiffening and PPARγ dysfunction, but direct causation has not been established. Establishing in vivo models with tunable tissue mechanics would help assess whether restoring normal mechanical properties rescues PPARγ activity and ameliorates lipid dysregulation. In addition, future studies should develop tissue‑specific approaches to dissect PPARγ mechanobiology in individual organs (e.g., liver versus adipose tissue) and generate real‑time reporters of PPARγ transcriptional activity to monitor its dynamic regulation under mechanical stress in living cells and animals. Validating the mechano‑PPARγ axis in disease‑relevant in vivo models (e.g., fibrosis‑prone, atherosclerosis‑prone, or diet‑induced obesity models) is essential to establish causation and guide therapeutic translation. Finally, for translational applications, on the one hand, developing selective PPARγ modulators with mechano‑mimetic properties could preserve insulin‑sensitizing effects while avoiding the weight gain and edema associated with traditional TZDs. On the other hand, exploring precision physical interventions, such as low‑energy shockwave therapy, vibration therapy, or wearable mechanical loading devices, may enable non‑invasive regulation of local fat deposition or metabolically dysfunctional tissues. Notably, recognizing exercise as a “natural mechanical intervention” and deeply investigating the molecular basis by which exercise remodels PPARγ function through mechanical signals may catalyze “exercise mimetics” development. Addressing these specific questions will not only consolidate the concept of PPARγ as a mechano‑metabolic transducer but also pave the way for a new generation of mechano‑based diagnostics and therapeutics for metabolic diseases.

In summary, this review establishes PPARγ as a central mechano‑metabolic transducer that integrates mechanical signals into lipid metabolic programs through multiple mechanotransduction pathways. By systematically dissecting the molecular basis of the mechanical signal‑PPARγ‑lipid metabolism axis, we provide a unified framework that explains how tissue‑specific mechanical microenvironments drive physiological homeostasis and, when aberrant, contribute to pathological remodeling in obesity, atherosclerosis, MASLD, and diabetes. The concept of PPARγ as a downstream transcriptional transducer distinguishes our work from existing reviews and opens new avenues for therapeutic intervention.

## Data Availability

Not applicable.

## Abbreviations

15d-PGJ₂: 15-Deoxy-delta(12, 14)-prostaglandin J(2)
2D: Two-dimensional
3D: Three-dimensional
9-HODE: 9-Hydroxyoctadecadienoic Acid
ABCA1: ATP-binding cassette transporter A1
ABCG1: ATP-binding cassette transporter G1
ACC: Acetyl-CoA carboxylase
Adipoq: Adiponectin
AF-1: Activation function-1
AF-2: Activation function-2
Akt: Protein kinase B
ALT: Alanine Aminotransferase
AMPK: AMP-activated protein kinase
AP-1: Activator protein-1
ASCs: Adipose-derived stem cells
AT1R: Angiotensin II receptor
BMP‑2: Bone morphogenetic protein 2
BMSCs: Bone marrow mesenchymal stem cells
C/EBP: CCAAT/enhancer binding protein
C/EBPα: CCAAT/enhancer-binding protein alpha
C/EBPβ: CCAAT/enhancer-binding protein beta
cAMP: Cyclic adenosine monophosphate
CDK5: Cyclin-dependent kinase 5.
CLA: Conjugated linoleic acid
COX-2: Cyclooxygenase-2
CPT1A: Carnitine palmitoyltransferase 1A
CREB: Cyclic-AMP response element-binding protein
CTSK: Cathepsin K
CYP27: Cytochrome P450 family 27
DBD: DNA-binding domain
EA: Electroacupuncture
ECM: Extracellular matrix.
ECs: Endothelial cells
EndMT: Endothelial-to-mesenchymal transition.
ERK: Extracellular signal-regulated kinase
EZH2: Enhancer of Zeste Homolog 2
FABP4: Fatty acid binding protein 4
FAK: Focal adhesion kinase
FAS: Fatty acid synthase
FFA: Free Fatty Acids
GelMA: Gelatin methacryloyl
GIP: Glucose-dependent insulinotropic polypeptide
GLIS2: GLIS family zinc finger 2
GLP-1: Glucagon-like peptide-1
GLUT-4: Glucose transporter type 4
GSK-3β: Glycogen synthase kinase 3 beta
HbA1c: Hemoglobin A1c
HDAC3: Histone Deacetylase 3
HDL-C: High-Density Lipoprotein Cholesterol
HUVECs: Human umbilical vein endothelial cells
IBD: Inflammatory Bowel Disease
IL-1β: Interleukin-1 beta
IL-6: Interleukin-6
IPF: Idiopathic pulmonary fibrosis
JNK: C-Jun N-Terminal Kinase
KLF2: Krüppel-like factor 2
LBD: Ligand-binding domain
LDHA: Lactate Dehydrogenase A
LMMS: Low-magnitude mechanical signals
lncRNA-MEG3: Long non-coding RNA maternally expressed gene 3
LXRα: Liver X receptor alpha
MAPK: Mitogen-Activated Protein Kinase
MASLD: Metabolic dysfunction-associated steatotic liver disease
miR-140-5p: MicroRNA-140-5p
MMP2: Matrix metalloproteinase 2
MPC: Mitochondrial Pyruvate Carrier
MSCs: mesenchymal stem cells
mTOR: mammalian Target of Rapamycin
NASH: Non-Alcoholic Steatohepatitis
NCT: National Clinical Trial
NEDD4: neural precursor cell expressed developmentally down-regulated protein 4
NF-κB: Nuclear Factor kappa B
OPG: Osteoprotegerin
OSS: oscillatory shear stress
PGC-1α: PPARγ co-activator-1α
PI3K: Phosphoinositide 3-kinase
PKA: protein kinase A
PKCα: protein kinase C alpha
PKM2: Pyruvate kinase M2
PPARα: peroxisome proliferator-activated receptor alpha
PPARγ: Peroxisome proliferator-activated receptor gamma
PPREs: Peroxisome-proliferator response elements
RANKL: receptor activator of nuclear factor-κB ligand
RhoA: Ras homolog family member
ROCK: Rho-associated protein kinase
ROS: Reactive oxygen species
Runx2: Runt-related transcription factor 2
RXR: retinoid X receptor
SCD1: stearoyl-CoA desaturase 1
SHRs: spontaneously hypertensive rats
Sirt1: Sirtuin 1
Smad: Sma- and Mad-related protein
SOX9: SRY-box transcription factor 9
SPPARγMs: Selective PPARγ Modulators
Src: proto-oncogene tyrosine-protein kinase
SRC-1: steroid receptor co-activator-1
T2D: type 2 diabetes
TAZ: transcriptional coactivator with PDZ-binding motif
TEAD: Transcriptional Enhancer Associate Domain
TG: triglyceride
TGF-β: transforming growth factor-beta
THRβ: Thyroid Hormone Receptor beta
TLR4: Toll-Like Receptor 4
TNF-α: Tumor necrosis factor-alpha
TRPV4: transient receptor potential cation channel subfamily V member 4
TSPCs: tendon stem/progenitor cells
TZD: thiazolidinedione
UCP1: uncoupling protein 1
VSMCs: vascular smooth muscle cell
XZF: Xiaozhi Fang
YAP: Yes-associated protein
